# A federated incremental blockchain framework with privacy preserving XAI optimization for securing healthcare data

**DOI:** 10.1038/s41598-025-21852-3

**Published:** 2025-10-30

**Authors:** Tanisha Bhardwaj, K. Sumangali

**Affiliations:** School of Computer Science Engineering and Information Systems, Vellore Institute of Technology, Vellore, Tamilnadu 632014 India

**Keywords:** Blockchain, Chaotic Bobcat optimization algorithm (CBOA), Decentralized learning, Explainable artificial intelligence (XAI ), Privacy preserving federated incremental learning blockchain (PPFILB ), Computational biology and bioinformatics, Engineering, Mathematics and computing

## Abstract

Federated learning (FL) has become more popular in the area of machine learning for protecting data privacy, its unique distributed data processing characteristics have garnered widespread attention. However, the implementation of FL faces many challenges, it can be difficult has to decide on a compromise between model security, data privacy, and system efficiency, often requiring the give up of efficiency for privacy, traceability, interpretability, and security. In this paper, privacy-preserving federated incremental learning blockchain-optimized explainable artificial intelligence (PPFILB-OXAI) leveraging the benefits of Blockchain, Federated Incremental Learning (FIL), and explainable artificial intelligence (XAI) with optimization. Chaotic Bobcat Optimization Algorithm (CBOA) is introduced to XAI for selecting most important features from the dataset. The CBOA mimics the instinctive behaviors of wild bobcats, incorporating a chaotic operator to randomly generate the population during the selection phase. It is inspired by the bobcat’s hunting tactics, particularly the approach and pursuit of prey. Throughout the algorithm iterations, the most optimal feature solution is gradually identified. The FIL algorithm is capable of adapting to increasing resources in real-time without the need for retraining, all while extracting meaningful patterns from the collective client side data. Meanwhile, Blockchain technology makes it possible to handle medical data securely and transparently, and XAI improves the clarity and understanding of model decisions. To coordinate client privacy protection, PPFILB-OXAI integrates the blockchain process, FIL, and privacy approach. It then uses an aggregate to filter out aberrant models. Lastly, Entropy Deep Belief Network (EDBN) has shown the ability to classify and identify attacks. PPFBXAIO provides the best performance on a breast cancer wisconsin and heart disease in terms of precision, recall, f-measure, accuracy, loss, latency, and throughput. Heart disease, the precision, recall, f-measure, and accuracy of the suggested system are 94.87%, 96.73%, 95.79%, and 95.71%, respectively. The precision, recall, f-measure, and accuracy of the suggested method for breast cancer wisconsin are 97.13%, 97.70%, 97.41%, and 96.84%, respectively.

## Introduction

There is a pressing requirement for sophisticated data mining algorithms as the amount and diversity of healthcare data keep growing to effectively analyze this information. To diagnose diseases, this type of study is essential, offering medical recommendations, and enhancing patient care. Machine learning, known for its strong computational power, has proven to be a valuable asset across various domains, including image analysis, natural language understanding, and the healthcare sector. Machine learning models typically need substantial quantities of training data to get enhanced precision, a critical factor in healthcare where outcomes can be lifesaving. Conventional centralized training approaches often involve aggregating vast amounts of data, which raises significant concerns about user privacy particularly when dealing with sensitive medical information.In most existing machine learning frameworks, end devices must send the gathered data for model training to a central server, which presents two major challenges. First, transmitting data can lead to high communication costs. Second, sharing raw data poses significant privacy risks, often discouraging data owners from uploading their information due to security concerns. Federated learning (FL), while also allowing collaboration model training across many end devices, which ensures the privacy of users, has surfaced as a solution to these problems^1^. In FL, clients will not have to provide raw data on a centralized server, in contrast to typical machine learning frameworks. Instead, only the model parameters derived from local data training are shared. This approach helps avoid privacy breaches associated with data transmission and also lowers communication overhead. In recent years, federated learning received a lot of academic attention and developed rapidly^2,3^. Nevertheless, the conventional FL framework continues to encounter certain challenges that can compromise the overall reliability of the system^4,5^.

**Fig. 1:**
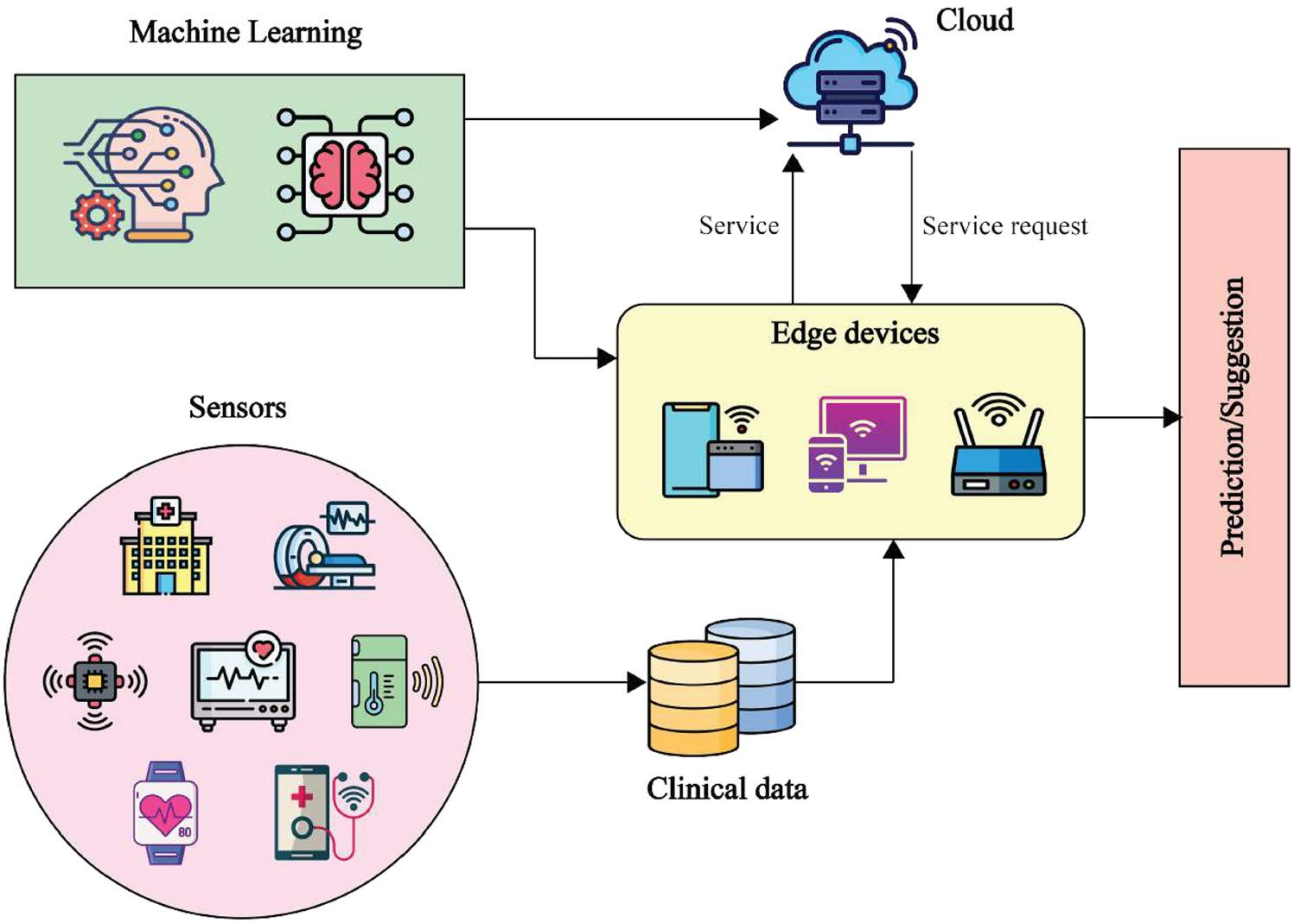
FL-based application for smart healthcare.

FL has been increasingly adopted in real-world scenarios, especially in sectors like healthcare, finance, transportation, and smart city development. Key functions in a FL system include maintaining the global model, choosing clients, and aggregating changes. These functions are handled by a central server. To perform aggregation, the clients must submit modifications; they will then receive the updated global data from the server model, which places significant strain on network bandwidth. Additionally, the performance of cloud-based servers is often influenced by cloud service providers dependability^6^. A central server may introduce bias into the global model by giving preferential treatment to certain clients. In addition, sensitive information from client updates or model corruption might result from an accessed server.Therefore, it is essential for FL to ensure the stability, equity, and security of the central server. In a standard FL-driven healthcare system (see Fig. 1), onboard sensors gather patient data, and several edge devices jointly execute the FL protocol. The resulting models then assess patients’ health and, if needed, trigger emergency services via the cloud. However, to get locally trained model updates, basic FL depends send the worldwide model to each device on a dependable central server, which remains a key limitation.

Blockchains immutable, collaboratively maintained, and traceable ledger can stand in for the central server, decentralizing FL coordination and guarding against single points of failure and unauthorized tampering.The fundamental concept is to eliminate the server entirely and distribute its aggregation and distribution duties across the participating client nodes. In this model, the blockchain itself acts as a decentralized repository underpinning the FL process^7,8^. In this approach, typical blockchain components correspond to the different phases of federated learning: each block signifies one training iteration, with the transactions within that block containing device-submitted model parameters for that cycle. Subsequently, all devices can access the most recent block to retrieve these parameters and update their local models accordingly. Considering these benefits, several blockchain-based federated learning approaches have been suggested for use in various domains, comprised of the Industrial Internet of Things (IoT), smart healthcare^9^ and smart homes^10,11^.

Blockchain-Based Federated Learning (BFL) has shown promise as a strategy for use in industries with strict privacy requirements. Leveraging blockchains strengths in identity authentication, decentralization, transparency, and data integrity, numerous studies have adopted blockchain technology as the foundational framework for FL. While blockchain enhances security while storing and updating models, it also successfully substitutes FL’s central server, it also brings new challenges to FL applications, including issues related to training speed, resource management, and communication latency.

AI-driven analytics has enhanced digital twins but increased is concerned about data security, privacy, and automated decision-making and transparency^12^. To tackle these challenges, it is essential to develop a complete platform integrating FL and blockchain technologies, and XAI, ensuring a digital twin ecosystem that is secure, scalable, and easy to interpret.Models may now be trained where the data comes from on edge devices through federated learning, a potent technique for distributed machine learning^13^. Explainable AI methods improve the transparency of these models by revealing how decisions are made. This decentralized setup not only protects user privacy by keeping raw data local but also enhances model robustness by incorporating a variety of datasets^13^.

This paper addresses the issues of efficiency for privacy, traceability, interpretability, and security. It has been solved by introducing a privacy preservation based Federated Incremental Learning (FIL), AI based blockchain framework, and classification methods for healthcare data. The major contribution of the work is described as follows,

*Integration of Privacy-preserving model with technologies*: Privacy-preserving model is integrated to FIL. FIL can constantly learn new knowledge and can save most of the previously known knowledge for increasing more privacy in the healthcare data.This method can mine the properties of the whole client-side samples while dynamically managing the growth in resources without retraining.

*Optimization with XAI based feature selection*: Chaotic Bobcat Optimization Algorithm (CBOA) is introduced to XAI for selecting the most important features from the dataset.

*PPFILB*XAI improves interpretability and increases model privacy, blockchain technology guarantees transparent and safe healthcare data administration. The PPFILB architecture offers a substantial protection in the FL process by preventing unwanted changes and guaranteeing data integrity.

*Attack detection and classification*: Entropy Deep Belief Network (EDBN)using Restricted Boltzmann Machines (RBMs) as its building blocks for disease diagnosis. In a breast cancer wisconsin and heart disease, results are assessed using metrics.

##  Literature review

Sezer et al.^14^ presented a PPFchain, a blockchain-based federated learning approach, protects sensor-IoT systems utilizing sampled Electrochemical Sensors (ECS) data. For blockchain networks in the IoT context, PPFchain offers a high-performance, low-cost, and lightweight architecture. To preserve efficiency while protecting off-chain fog nodes’ privacy of users and data, the architecture combines cryptographic approaches with federated learning models. Additionally, conventional blockchain frameworks are evaluated across several performance metrics within a distributed system, including event-driven and storage-based smart contracts. The findings demonstrate that PPFchain delivers improved accuracy, efficiency, and security.

Li et al.^15^ developed a user-friendly and privacy-focused system called Alzheimer’s Disease Detector (ADDetector) with leveraging IoT devices and security technologies. ADDetector gathers users’ audio data through IoT devices commonly installed in smart homes and enhances detection accuracy by using advanced topic-based elements of language. To address privacy risks at the data, feature, and model levels, the user, client, and cloud levels are all part of the unique three-tier architecture that ADDetector uses to secure privacy. The ADDetector system employs a FL approach to guarantee that users maintain control over their unprocessed data and to protect the classification model. To further strengthen feature privacy, a Differential Privacy (DP) mechanism is incorporated. Additionally, to protect client data and the cloud’s model aggregation process, the FL framework provides special asynchronous privacy-preserving aggregation architecture.

Ngan Van et al.^16^ presented a PriFL-Chain as a privacy-preserving framework that leverages data resources from data owners through machine learning models while safeguarding their privacy. In this approach, users may exchange just locally trained models, not their raw data, because of DP’s integration with federated learning for training models. Additionally, to provide transparency, the blockchain records user contributions made throughout the system. In minimizing the core server’s workload and lowering data transfer costs, Mobile Edge Computing (MEC) and the InterPlanetary File System (IPFS) provide system flexibility. According to experimental results, federated learning, blockchain, IPFS, and MEC may be integrated to effectively exploit a variety of community data resources, reduce the expense of training machine learning models, and provide robust privacy protection.

Madill et al.^17^ proposed a scalable blockchain-based approach to federated learning (ScaleSFL). Off-chain federated learning is distinct, which improves compatibility, focusing on verifying model updates rather than managing the entire federated learning process. To confirm the approach’s viability, ScaleSFL was developed as a Hyperledger Fabric proof-of-concept prototype. Performance assessments were conducted using Hyperledger Caliper benchmarking tools. The results indicate that sharing can enhance validation performance in a linear manner while maintaining both efficiency and security.

Cui et al.^18^ introduced Blockchain-based Federated Learning (BCFL) framework that accelerates training by compressing communications to improve efficiency. BCFL increase the convergence rate for nonconvex loss functions. They optimization task is introduced to maximize the accuracy of model, and reduce the convergence-rate training loss within a predetermined time budget by modifying the ratio of compression to block production. Finally, utilizing the CIFAR-10 and FEMNIST datasets, they conducted comprehensive trials to confirm BCFL’s performance.

Lo et al.^19^ suggested increasing accountability and equity in the learning process, a federated learning framework built on the blockchain. To maintain accountability, the architecture incorporates a smart contract-based register for the provenance of data and models. A weighted fair data selection mechanism was used to further encourage fairness in the training dataset. Results showed that the method was successful in improving both accountability and fairness when tested on a COVID-19 X-ray detection task. Furthermore, regarding the generality and accuracy of the model, the suggested approach performed better than the conventional federated learning configuration.

Miao et al.^20^ introduced a blockchain-based Privacy-preserving Byzantine-robust Federated Learning (PBFL) system reduces central server influence and protects against malicious clients. The method uses cosine similarity to identify and evaluate harmful gradients submitted by attackers. Additionally, data aggregation is secure with complete homomorphic encryption. Finally, a blockchain system is implemented to promote transparency and enforce regulatory compliance. The suggested technique ensures convergence while protecting privacy, according to formal analysis. Comprehensive experiments across various datasets show that the PBFL approach is both robust and efficient. Notably, even with a small root dataset, PBFL achieves performance comparable to Federated Stochastic Gradient Descent (FedSGD).

Tian et al.^21^ presented a decentralized deep federated learning (RPDFL) training method is both secure and private. Ring-Allreduce data sharing system is introduced for federated learning to enhance communication efficiency during RPDFL training. Additionally, the use of the Chinese remainder theorem improved the parameter distribution procedure. This guarantees the resilience of RPDFL under the RingAllreduce data sharing paradigm by enabling healthcare edge devices to stop training without increasing the risk of data leaking. Security evaluations demonstrate that RPDFL offers provable security. Experimental findings reveal that RPDFL outperforms traditional federated learning techniques in both model accuracy and convergence speed, giving it an excellent fit for digital healthcare applications.

Guduri et al.^22^ introduced federated learning blockchain-based lightweight encryption technique to Electronic Health Records (EHR) scalability and trust. A decentralized cloud environment is used to store the EHR data. For federated learning length, the system ensures that all data is completely secured. To control the flow of EHR data, sensors and data consumers continually produce smart contracts. Federated learning when combined with use of the effective proxy re-encryption technique ensures user and data owner privacy while contracts are being executed.

Ouyang et al.^23^ presented a novel federated learning privacy architecture that uses smart contracts and blockchain to allow completely private, off-chain collaborations while conducting learning on-chain. The framework comprises two components secure peer-to-peer identification and confidential federated learning each governed by its own scalable smart contract. Built on Ethereum and using IPFS for storage, the framework has been implemented, and its performance has been thoroughly evaluated. The proposed framework maintains reasonable collaboration costs while providing benefits in privacy, security, and decentralization. Additionally,it has the potential to provide autonomous federated learning among machine clusters composed of IoT devices, with automated on-chain identification when combined with radio frequency identification (RFID) technology.

Wang et al.^24^ proposed a BCFL incentive mechanism to assist the Model Owner (MO), publishes the BCFL task in allocating incentives for clients. In response, stackelberg game architecture with two stages is used by client to determine how much processing resources to give to each subtask. The most effective strategies for each party are determined by resolving two sequential optimization problems derived from the game model, which is analyzed by considering the utility of clients and the MO. Additionally, acknowledging that clients’ local training information may be private, analytical solutions are added to the game model to handle situations when there is incomplete information.

Yazdinejad et al.^25^ presented a new Auditable Privacy-Preserving Federated Learning (AP2FL) approach for healthcare electronic. AP2FL greatly lowers the danger of data leakage by securing the client and server-side training and aggregation phases using Trusted Execution Environments (TEE). Non-Independent and Identically Distributed (Non-IID) data is managed by combining the Active Personalized Federated Learning (ActPerFL) approach with Batch Normalization (BN) approaches to aggregate user updates and find data commonalities. To enable the global model to adjust to different types of data and distributions, AP2FL additionally includes an audit system that measures contribution of each client to the process of federated learning. The integrity, openness, equity, and resilience of the FL process are ensured by this feature. According to experimental results, AP2FL outperforms existing methods in terms of accuracy and successfully prevents privacy leaks.

Hu et al.^26^ proposed FL-HMChain with healthcare and medical data collaboration which protects privacy by using blockchain technology and FL. Three levels form the system: data management, data application, and data extraction and storage. To securely manage data storage, transportation, the process, and access while ensuring real-time efficiency, dependability, and data integrity, the system is specifically made for healthcare and medical data. It develops a blockchain specifically for this purpose. An enhanced consensus mechanism for selecting master nodes is proposed to identify and deter malicious actions, ensuring the collaborative model training process’s dependability and integrity. The Proposed model has increased results when compared to Convolutional Neural Network (CNN) with an average increase of 7.00% by Accuracy (ACC) and 4.70% by Area Under the Curve (AUC). In addition, the potential of breaches of privacy between local and global models is greatly reduced by the federated learning system’s blockchain-enabled parameter transfer mechanism.

Tian et al.^27^ proposed a Privacy-Preserved and Efficient Federated Learning (PEFL) framework incorporating blockchain. Blockchain and differential privacy methods are introduced to ensure coordinated privacy protection among clients and an aggregation-side detection algorithm is introduced to identify and exclude abnormal model parameters, thereby defending against poisoning attacks. Committee-based model validated fault-tolerant federation (MFF) is introduced to stability effectiveness goals, adjust the server, and preserve the dependability of the process. Experiments conducted on the MNIST and CIFAR10 datasets, along with comparisons to standard federated learning approaches, show that PEFL offers stronger protection against multiple attack types. Additionally, it delivers improved training efficiency while safeguarding privacy.

Asad and Otoum^28^ created a novel Blockchain-Based Framework for Privacy-Preserving Federated Learning (BPPFL). As the blockchain records transactions and model changes in an unchangeable ledger, this architecture offers secure participant authentication while protecting against internal and external adversaries.

BPPFL framework maintains is highest accuracy and secure privacy while significantly reducing computing and communication costs when compared to existing methods. For sectors like healthcare, banking, and the IoT, security and dependability of federated learning systems is increased when compared to other methods.An overview of existing approaches, including their benefits and drawbacks, is given in Table 1

**Table 1:** Literature review of existing methods.

Author and citation	Approach	Blockchain platform	Key technologies	Advantages	Disadvantages
Sezer et al.^14^	PPFchain	Blockchain	sensor-IoT-based architectures	Precision, effectiveness, and improved security are provided by PPFchain	Real-time data traceability, availability, and low latency
Li et al.^15^	ADDetector, DP	NA	IoT, FL based scheme	The aggregation procedure among clients and the cloud should be protected by a FL-based system	Data transmission leakage of the model and raw data
Ngan Van et al.,^16^	PriFL-Chain, DP	Blockchain	FL based scheme, MEC	Minimize the expense of ML model training, efficiently safeguard privacy, and make use of diverse data sources	Large-scale deployment of IoT devices is difficult and expensive
Madill et al.^17^	ScaleSFL	Blockchain-based sharing- Hyperledger Fabric	FL	Maintain efficiency and security while increasing validation performance linearly	Provide the appearance of malevolent system assaults
Cui et al.^18^	BCFL, smart contract	Blockchain	FL	Minimize the training loss, and training time of classifier	Communication load of BFL is higher
Lo et al.^19^	Trustworthy federated learning based on blockchain	Blockchain	FL	Enable accountability and improve fairness	The amount of samples every client has during classification decreases as the number of customers declines
Miao et al.^20^	PBFL	Blockchain	FL	Achieves convergence and provides privacy protection.	The suggested model has not examined the communication impose of PBFL
Tian et al.^21^	RPDFL, Ring-All reduce-based data sharing	Blockchain	FL	According to security studies, RPDFL has improved accuracy, convergence, and is provably secure	Real-time applications are not supported
Guduri et al.^22^	blockchain-based lightweight encryption strategy	Blockchain	Cloud computing	Proposed system very effective proxy re-encryption mechanism with FL	Strive for credibility and embrace responsible AI concepts
Ouyang et al.^23^	FL based on blockchain	Blockchain	RFID, FL, IoT	Benefits related to decentralization, privacy, and security.	Doesn’t support for large-scale applications
Wang et al.^24^	BCFL, two-stage Stackelberg game	Blockchain	FL	Clients in determining the lesser computational time.	Malicious clients and servers have the ability to poison federated learning systems
Yazdinejad et al.^25^	AP2FL	NA	ActPerFL	Robustness, fairness, openness, and integrity of the FL process. Improved precision and successful removal of privacy leaks	Inadequate privacy protection for larger datasets
Hu et al.^26^	FL-HMChain, FL-CNN-HMChain	Blockchain	FL	Ensuring that the cooperative model training procedure is generally dependable and trustworthy. The suggested model significantly reduces the probability of privacy leaking	More attention must be paid to developing more accurate and dependable prediction models for the medical field
Tian et al.^27^	PEFL, DP	Blockchain	MFF	PEFL demonstrates better defense against various attack models. Proposed system achieves higher training efficiency while ensuring privacy security	Less support for efficiency, and reliability in data sharing
Asad and Otoum^28^	BPPFL	Blockchain	IoT, FL	Reduces computation and communication overhead. Highest model accuracy and robust privacy guarantees	Enhancing throughput and reducing latency in the blockchain’s infrastructure

##  Proposed methodology

**Fig. 2:**
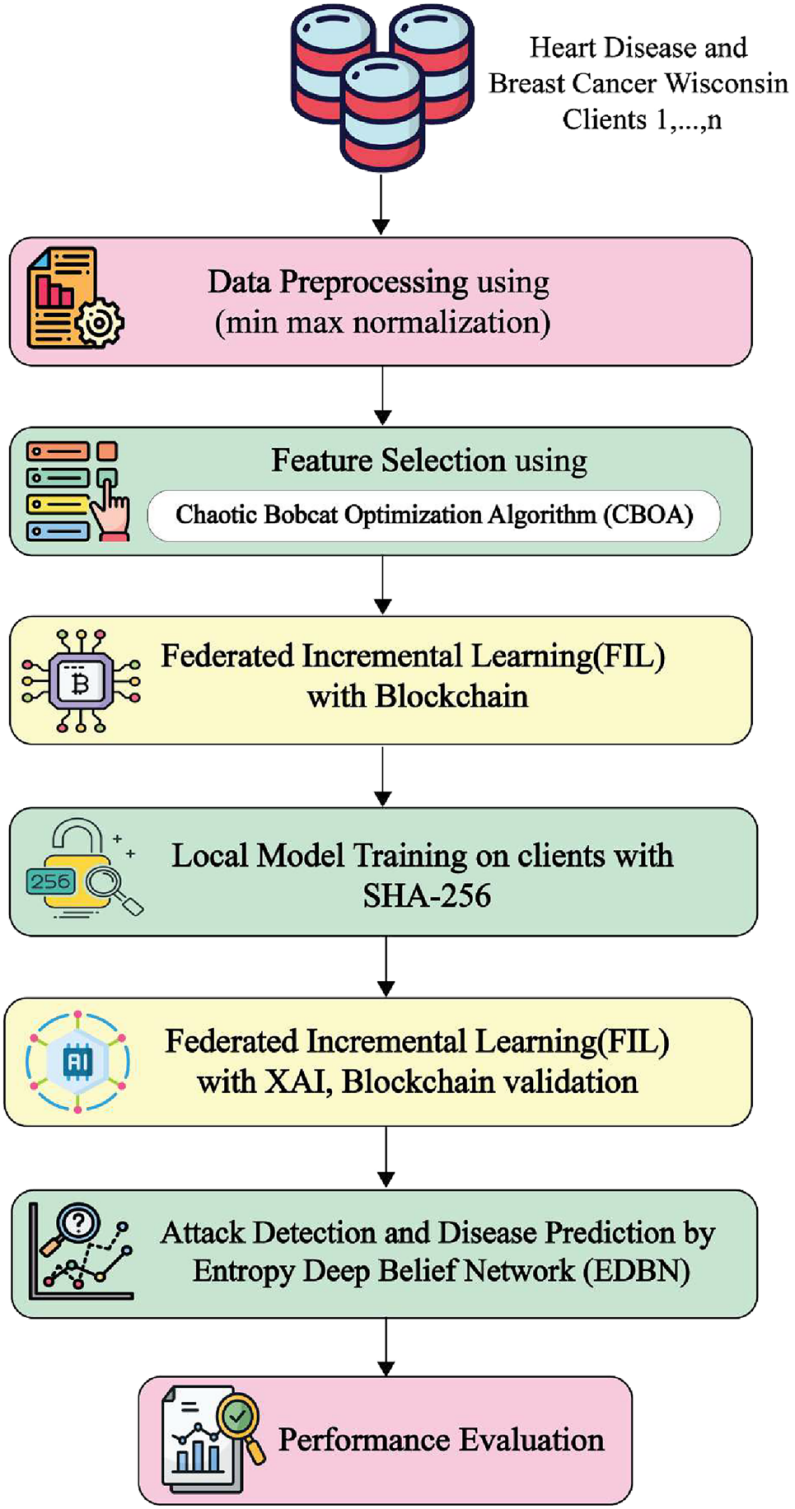
Workflow of proposed PPFILB-OXAI framework.

Proposed is the integration of Blockchain, FIL, and XAI with optimization. Chaotic Bobcat Optimization Algorithm (CBOA) is introduced to XAI for selecting most important features from the dataset. This method can mine the properties of the entire client-side samples while dynamically managing the growth in resources without retraining. It combines the procedure of blockchain, FIL, privacy technique to prevent poisoning attempts by filtering out unusual model parameters using an aggregation approach and organizing client privacy protection. While XAI improves interpretability, blockchain technology guarantees transparent and secure healthcare data administration. Finally, assault detection and categorization have been carried out by Entropy Deep Belief Network (EDBN). Figure 2 illustrates the suggested model’s general procedure.

###  Dataset collection

For this study, the breast cancer wisconsin and heart disease were gathered from Kaggle to replicate a distributed setting where every client has their own dataset. Heart illness dataset is collected from https://www.kaggle.com/datasets/johnsmith88/heart-disease-dataset and Cleveland, Hungary, and Switzerland. It includes 76 attributes in total, among which only 14 are commonly used in published studies. The“target”attribute indicates whether the patient has heart disease, where a value of 1 indicates the prevalence of the disease and a value of 0 indicates its absence. Breast Cancer Wisconsin dataset is collected from https://www.kaggle.com/datasets/uciml/breast-cancer-wisconsin-data.

**Fig. 3:**
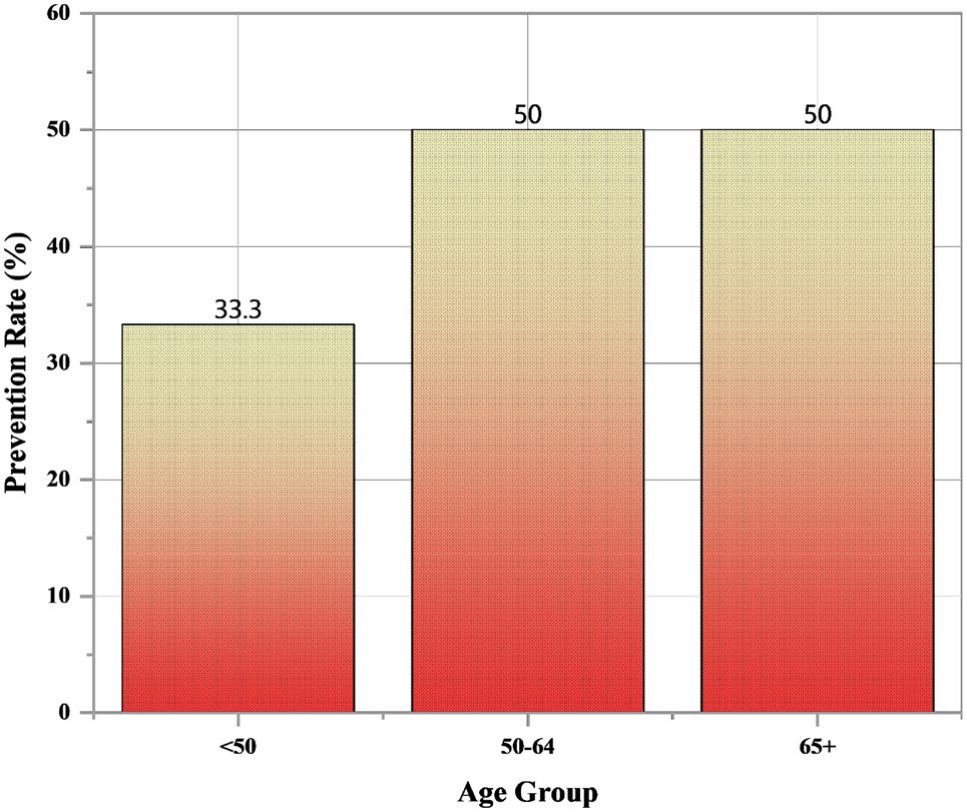
Preventive effect by age group.

Figure 3, dataset included individuals aged between 40 and 74 years, with a mean age of approximately 60 years. The sex distribution was  65% male (coded as 1) and  35% female (coded as 0). Clinical and demographic variables included chest pain type (cp), resting blood pressure (trestbps), serum cholesterol (chol), fasting blood sugar (fbs), resting electrocardiographic results (restecg), maximum heart rate achieved (thalach), exercise-induced angina (exang), ST depression induced by exercise (oldpeak), the slope of the ST segment (slope), number of major vessels colored by fluoroscopy (ca), thalassemia type (thal), and disease presence (target).

To examine whether the preventive strategy (“Prevent Attack”) was more effective in younger individuals, grouped participants into three age categories like < 50 years, 50-59 years, and 60 years. Preliminary results indicated a higher proportion of disease prevention in the < 50 years group compared to older age groups, suggesting that early intervention may offer stronger protective benefits against disease onset.

###  Min-max normalization in data preprocessing

Min-Max normalization is introduced to a technique in classifier. The new dataset undergoes linear scaling to map the feature values into a defined range, often in the range of 0–1. To achieve this, determine each feature’s lowest and maximum values, and then proportionately alter all other values. It adjusts the remaining values in relation to their original distribution places, changing the minimum value to 0 and the highest to 1. It uses Eq. (1) to determine the normalized value ($x'$).

1 $$\begin{aligned} x' = \frac{x - \min (x)}{\max (x) - \min (x)} \end{aligned}$$

Min(x) is the feature minimum value and max(x) is its maximum value when the original value is represented by x.

###  CBOA based feature selection

Feature selection is essential to increase the accuracy and effectiveness of disease prediction. CBOA method, applies to direct the population updates in the search space, using inspiration from bobcats’ natural hunting activity. This approach mimics the bobcat’s hunting behavior, where the animal initially locates the prey and moves closer to it. It then waits for the right moment to launch an ambush and eventually captures the prey after a chase. Based on this behavior, the bobcat’s movement during hunting can be divided into two phases: (i) locating and approaching the prey, and (ii) pursuing and capturing it. The CBOA framework is modeled after the natural hunting behavior of bobcats. Each iteration has two primary stages that update the positions of potential solutions in the feature space: (i) exploration, which simulates the bobcat’s movement as it approaches its target, and (ii) exploitation, this captures the best aspects by imitating the bobcat’s position modifications during its final search. The subsequent sections provide a detailed explanation of each phase of the CBOA update process^29^,

*Initialization:* The CBOA method operates as a population-based optimization technique, capable of identifying optimal feature subsets through an iterative search process conducted by its agents within the feature selection domain. The bobcat’s natural habitat serves as a metaphor for the problem-solving the area in the CBOA design, and each bobcat’s position within this space symbolizes a potential feature configuration in the solution search. In the CBOA, each bobcat symbolizes an individual within the population size of 30, with its position representing a potential feature subset for classification. Mathematically, this position is modeled as a vector, where each element denotes a specific decision variable. Collectively, Eq. (2) illustrates the population of the algorithm, which is made up of these bobcats, may be represented as a matrix. Equation (3) generates the bobcats’ beginning positions inside the feature selection space at random.

2 $$\begin{aligned} & \textbf{X} = \begin{bmatrix} \textbf{X}_1 \\ \vdots \\ \textbf{X}_i \\ \vdots \\ \textbf{X}_N \end{bmatrix}_{N \times m} = \begin{bmatrix} x_{1,1} & \cdots & x_{1,d} & \cdots & x_{1,m} \\ \vdots & \ddots & \vdots & \ddots & \vdots \\ x_{i,1} & \cdots & x_{i,d} & \cdots & x_{i,m} \\ \vdots & \ddots & \vdots & \ddots & \vdots \\ x_{N,1} & \cdots & x_{N,d} & \cdots & x_{N,m} \end{bmatrix}_{N \times m} \end{aligned}$$

3 $$\begin{aligned} & x_{i,d} = lb_d + (ub_d - lb_d) \end{aligned}$$

where *r* is a random integer in the interval [0, 1], where *m* is the number of decision variables and *N* is the number of bobcats, $X_i$ is the *i*th bobcat (feature solution), $x_{i,d}$ is the feature selection space’s *d*th dimension, and *X* is the BOA population matrix. The *d*th lowest and greater boundaries dimension are denoted by *lbd* and *ubd*. The model can assess the accuracy of each bobcat’s position, which constitutes a feature selection for the classification. Equation (4) allows us a vector representation of the set of precision.

4 $$\begin{aligned} F = \begin{bmatrix} F_1 \\ \vdots \\ F_i \\ \vdots \\ F_N \end{bmatrix}_{N \times 1} = \begin{bmatrix} F(X_1) \\ \vdots \\ F(X_i) \\ \vdots \\ F(X_N) \end{bmatrix}_{N \times 1} \end{aligned}$$

F denotes the vector of accuracy scores, and $F_i$ represents the accuracy achieved by the ith bobcat. These accuracy evaluations serve as metrics for assessing the quality of each candidate solution in CBOA. Consequently, the individual with the highest accuracy value is regarded as the top-performing bobcat, while the one with the lowest accuracy value is deemed the least effective. In the CBOA framework, the positions of the bobcats within the feature selection space are revised in each iteration, leading to corresponding updates in candidate solutions and their associated accuracy scores. As a result, the top-performing bobcat must be re-evaluated and updated in every iteration based on the latest accuracy comparisons.

#### Tracking and moving towards prey (exploration phase)

In the initial phase of CBOA, the positions of the individuals in the population are adjusted by simulating the tracking and approach behavior of bobcats as they hunt prey. This modeled movement results in significant shifts in their positions within the feature selection space, thereby enhancing CBOA’s exploration capability for conducting a global search. In this mechanism, each bobcat identifies other population members with higher accuracy scores as potential prey. Each bobcat’s list of potential prey is defined using Eq. (5).

5 $$\begin{aligned} CP_i = \{X_k: F_k < F_i \text { and } k \ne i\}, \text { where } i = 1, 2, \ldots , N, \, k \in \{1, 2, \ldots , N\} \end{aligned}$$

The collection of potential prey provides for the ith bobcat is denoted by $CP_i$, and the classification accuracy of the member of the population that has a precision value higher than the ith bobcat is indicated by $F_k$. The CBOA framework, each bobcat randomly picks one prey from its candidate set and targets it. By emulating the bobcat’s motion toward its prey, using Eq. (6), the algorithm determines a new position for each agent.If this updated position leads to a higher accuracy is evaluated using Eq. (7) with supplants the agent’s previous position

6 $$\begin{aligned} & x_{i,j}^{P1} = x_{i,j} + (1 - 2r_{i,j}) \cdot (SP_{i,j} - I_{i,j} \cdot x_{i,j}) \end{aligned}$$

7 $$\begin{aligned} & X_i = {\left\{ \begin{array}{ll} X_i^{P1}, & F_i^{P1} <= F_i \\ X_i, & \text {else} \end{array}\right. } \end{aligned}$$

where, $SP_i$ is the prey that the bobcat has selected, $SP_{i,j}$ is the dimension of its *j*th, $X_s$ based on the CBOA’s examination phase, the new position determined for the *i*th bobcat, $x_{i,j}^{P1}$ is the measure of its *j*th, $F_i^{P1}$ is the value of its purpose function, $r_{i,j}$ are randomized values drawn from the [0, 1] interval, and $I_{i,j}$ are selected at random numbers, such as 1 or 2.

#### Chasing to catch prey (exploitation phase)

**Fig. 4:**
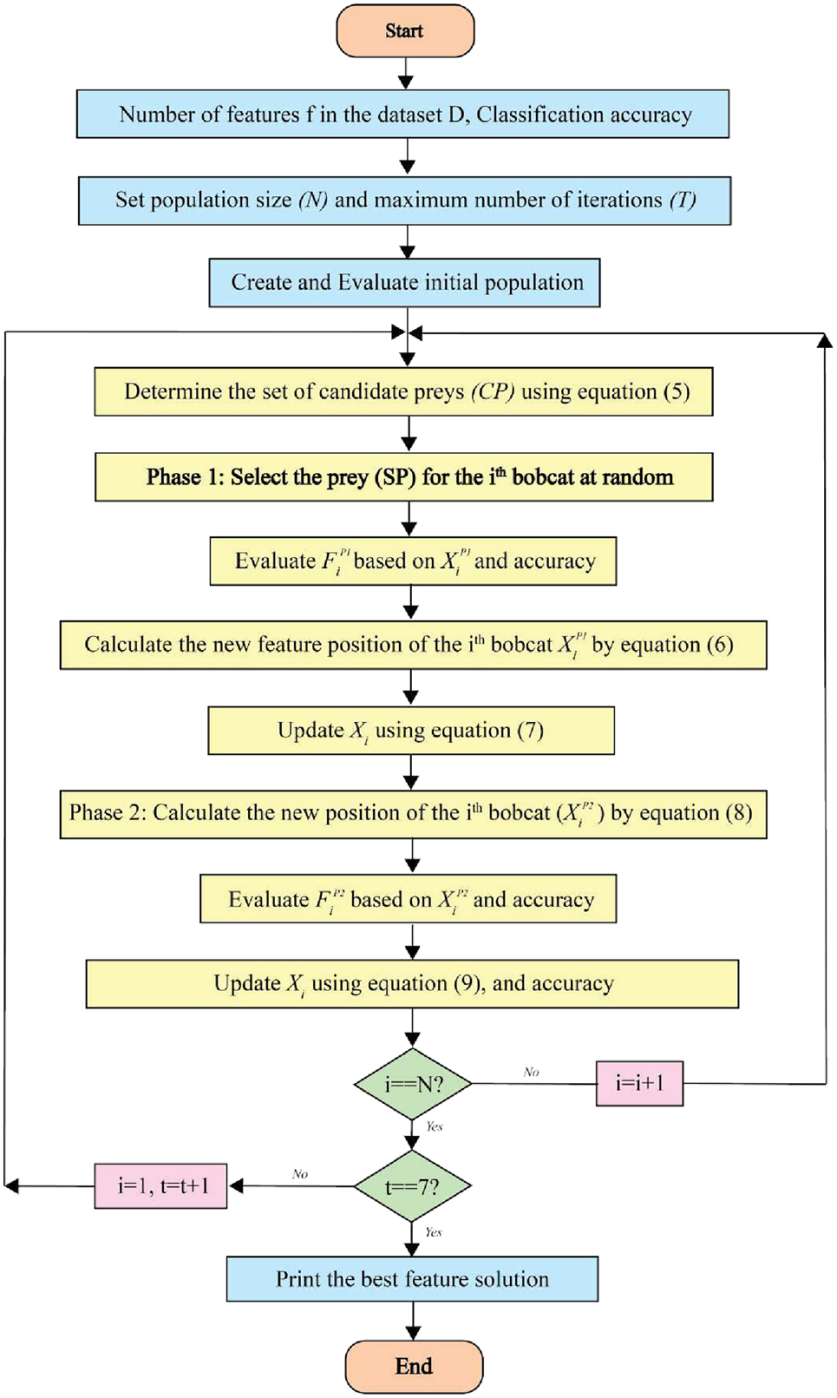
Flowchart of CBOA.

CBOA second phase, candidate solutions are fine-tuned by mimicking the bobcat’s close-range pursuit and capture of prey. Because this chase occurs in the immediate vicinity of the hunt, only slight adjustments are made to each agent’s position within the feature space, thereby strengthening CBOA exploitation capability for effective local search. In the CBOA framework, the new position of each member is determined by imitating the movements of the bobcat in the pursue phase near the hunting area, as described by Eq. (8). If this updated position results in better accuracy, it replaces the member’s former position following Eq. (9).

8 $$\begin{aligned} & x_{i,j}^{P2} = x_{i,j} + \frac{1 - 2r_{i,j}}{1 + t} \cdot x_{i,j} \end{aligned}$$

9 $$\begin{aligned} & X_i = {\left\{ \begin{array}{ll} X_i^{P2}, & F_i^{P2} <= F_i \\ X_i, & \text {else} \end{array}\right. } \end{aligned}$$

where $X_i^{P2}$ is the new *i*th position bobcat determined by the anticipated CBOA’s exploitation phase, $x_{i,j}^{P2}$ is its *j*th dimension, $F_i^{P2}$ is its accuracy, *t* is the number of iterations, and $r_{i,j}$ stands for random numbers from the interval [0, 1]. CBOA approach, the exploration and exploitation phases are updated and then the next iteration of the method is conducted performed using the revised positions and accuracy values of the bobcats. This update cycle continues for each subsequent iteration until the algorithm reaches its final iteration, guided by Eqs. (5)–(9). In each iteration, the best feature selection found up to that point is updated and stored. Once the algorithm completes all iterations, the top feature set identified throughout the process is provided as the final subset. If the optimal features are not found by the maximum number of iterations, the crossover operation is repeated until a better offspring replaces the weakest individual in the population or a predefined iteration limit is reached. The steps for implementing CBOA are illustrated in the flowchart in Fig. 4, and Algorithm 1 describes its pseudocode in complete.

**Algorithm 1:**
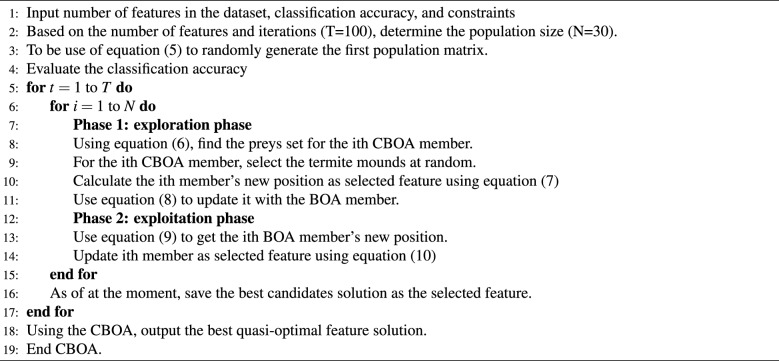
CBOA feature selection

### Privacy-preserving federated incremental learning blockchain optimized explainable artificial intelligence (PPFILB-OXAI)

In this section discuss the details of FBXIAO framework. FL and the deployment of AI models on individuals allow for user collaboration while protecting data privacy. Chaotic Bobcat Optimization Algorithm (CBOA) is introduced to XAI for selecting most important features from the dataset and it can improve the model’s performance by optimizing the federated averaging hyperparameters.While XAI improves interpretability, blockchain technology guarantees transparent and secure healthcare data administration.

#### Federated incremental learning (FIL)

To train a shared deep learning model, many clients could collaborate together using FL decentralized methodology, which protects their local data privacy^30^. In FIL settings, clients might not have sufficient storage capacity to hold all the data^31^. FIL initially distinguishes between two types of tasks for new obtained data when addressing a classification task:

1. *Class-incremental task*: Since the newly acquired data contains different labels than the earlier data, the data’s label space is expanding.
2. *Domain-incremental task*: The new data doesn’t alter the data’s label space, but compared to the previous data, there is a domain change. Assuming that each task’s data in clients is not independently and identically distributed (IID), it is therefore assumed that there is data heterogeneity.IID, each client’s local data follows similar to the global dataset’s distribution. Dataset is partitioned by randomly shuffling the dataset and evenly distributing samples.

In the normal IL, in a series of streaming tasks, a model learns (non-federated environment) $\{T_1, T_2, \cdots , T_n\}$ where $T_t$ denotes the *t*th task of the dataset. Here $T_t = \sum \limits _{i=1}^{N_t} (x^{(i)}_{t}, y^{(i)}_{t})$, which has $N_t$ pairs of sample data $x^{(i)}_{t} \in X_t$ and corresponding label $y^{(i)}_{t} \in Y_t$. Let us consider $X_t$ and $Y_t$ to show the *t*-th task domain and label space $|Y_t|$ classes and $Y = \bigcup \limits _{t=1}^{n} Y_t$ where *Y* is denoted as the total courses actually presented. Similarly, $X = \bigcup \limits _{t=1}^{n} X_t$ to represent the complete domain space for all tasks throughout existence.

Examine two categories of IL situations: $X_1 = X_t, \forall t \in [n]$ all tasks share the same domain space since it is a class-incremental task. The number of classes may vary as the learning task sequence presents itself, for example, $Y_1 \ne Y_t, \forall t \in [n]$. (2) Domain-Incremental Task: $Y_1 = Y_t, \forall t \in [n]$ implies that all tasks have the same number of classes. The client’s area and the dissemination of data are subject to modification, i.e., $X_1 \ne X_t, \forall t \in [n]$, they must simultaneously learn the new task.

Additionally, consider federated IL. In training assume that client *K* is limited to accessing local private streaming jobs in a global model for *K* clients. To train a global model is the objective $w^t$ across all *t* tasks $T^t = \{\sum _{n=1}^t \sum _{k=1}^K T^n_{k}\}$, which, even if customers are able to remember all samples from previous assignments, may be stated as follows.

10 $$\begin{aligned} w^t = \arg \min _{w} \sum _{k=1}^{t} \sum _{n=1}^{K} \sum _{i=1}^{N_k^n} \frac{1}{|T^t|} \; \mathscr {L} \left( f_{w_k} \left( x_{k,n}^{(i)} \right) , \; y_{k,n}^{(i)} \right) \end{aligned}$$

Where $\ell (\cdot )$ is the cross-entropy loss and $f_{wk}(\cdot )$ is the model $w_k$’s output in client *k*. Afterward, partial samples are cached by each client for replaying because of inadequate storage on common devices. This implies that each client must cache *M* samples and can only store a total of *M* samples.

11 $$\begin{aligned} & w^t = \arg \min _{w} \sum _{k=1}^{K} \sum _{i=1}^{M} \frac{1}{|T_{k,\text {local}}^t|} \left( f_{w_k} \left( \tilde{x}_{k,t}^{(i)} \right) , \tilde{y}_{k,t}^{(i)} \right) \end{aligned}$$

12 $$\begin{aligned} & {T_{k,\text {local}}^t}= \sum _{i=1}^{M} \left( \tilde{x}_{k,t}^{(i)}, \tilde{y}_{k,t}^{(i)} \right) \end{aligned}$$

**Fig. 5:**
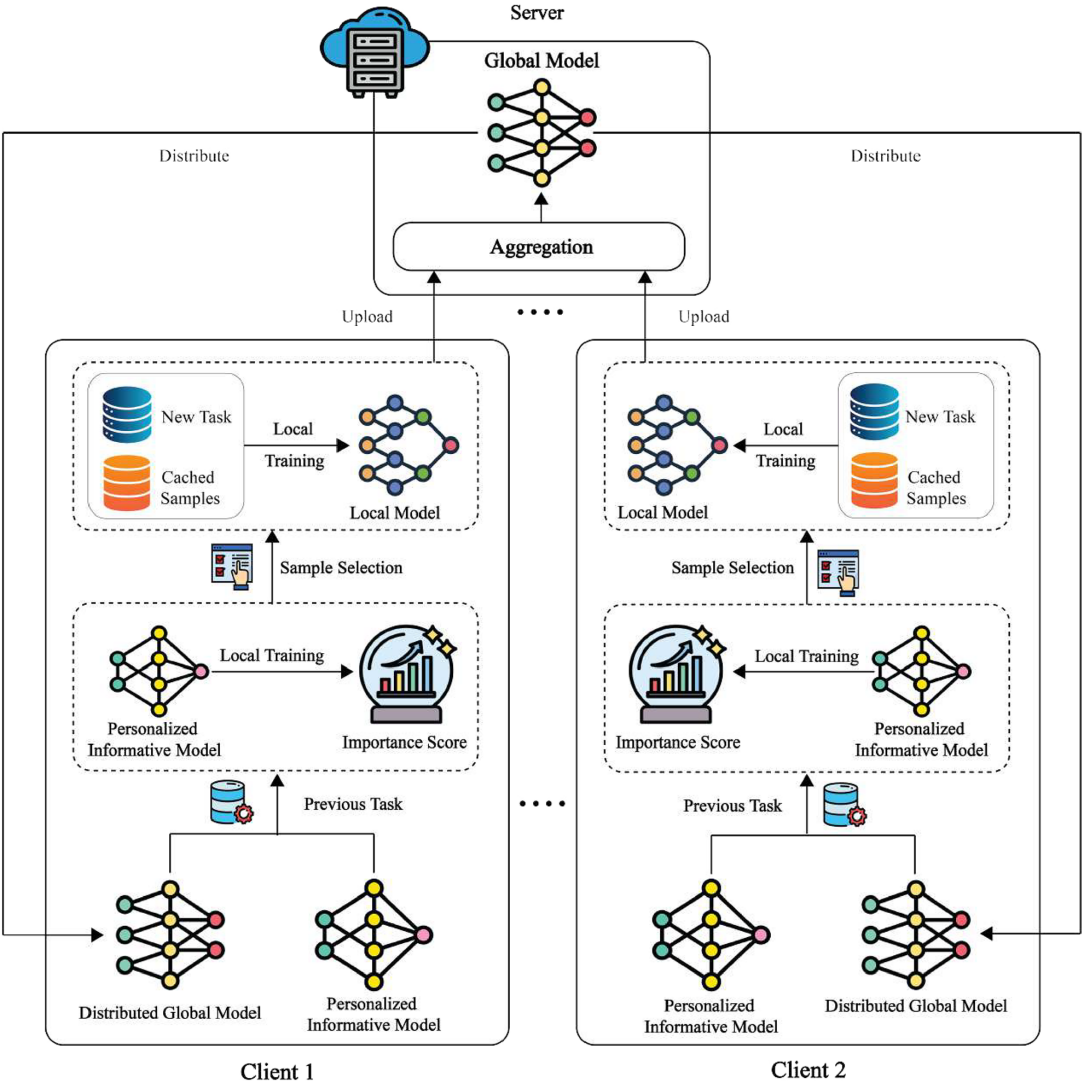
Illustration of the FIL framework.

More specifically, upon the arrival of a new task, each client initially stores a selection of previous samples determined by their global and local significance. The client then uses the data from the latest assignment in addition to these cached samples to train its local model. When a new task is provided, FIL concentrates on determining the significance of the samples and arranging for clients to preserve important prior samples in the constrained local storage, as shown in Fig. 5. The server then aggregates all from all participating clients. Clients use their own private task data to update their local models throughout each communication period. Through utilizing instruction from both each client must train a new informative model on its cached samples to complete new tasks, using both the global model and its own local model.

Each client starts by utilizing the distributed global model and previous local data to update its customized informative model when a new task is received. Next, samples are chosen for caching based on their importance scores, which are computed using the revised customized model. Finally, every client uses the cached samples from earlier tasks in addition to the recently received work to train its local model.

In FIL with Non-IID data across clients, determining sample importance should consider both the relevance within the local dataset and the relationship to the global dataset shared among clients. Clients datasets differ in data distribution, generally each client’s gets 10% of samples for each class.This ensures more effective training using cached samples. In typical FL, the common global model and their own local model alone, which stand for local and global knowledge, respectively, are available to clients. The proposed approach involves computing two separate importance scores one from combining the local and global models, as well as using these scores to decide which samples to cache for future reuse. This concept can be extended by incorporating the following features: (1) the creation of the global model involves merging the local models of the participating clients, and its gradient norm may be computed locally without the global model having to be directly trained. (2) When assessing sample importance, a control system should be set in place to manage the proportion of local to global information.

To achieve these objectives, each client is equipped with a Personalized Informative Model (PIM) that incorporates knowledge from regional and worldwide models. A ratio factor is employed to control the balance between local and global contributions. The samples’ gradient norms are noted during the PIM update and used for determining their significance ratings, effectively capturing both local and global perspectives. In *s* iterations, clients *k* get the global model $w^{t-1}$ and update PIM $v_{k}^{t-1}$ with local samples $T_{k,\text {local}}^{t-1}$ after receiving the *t*th new task.

13 $$\begin{aligned} u_{k,s}^{t-1} = v_{k,s-1}^{t-1} - \eta \left( \sum _{i=1}^{M} \nabla \ell \left( f_{v_{k,s-1}^{t-1}} \left( \tilde{x}_{k,t-1}^{(i)} \right) , \tilde{y}_{k,t-1}^{(i)} \right) + q(\lambda )\left( v_{k,s-1}^{t-1} - w^{t-1} \right) \right) \end{aligned}$$

Using $q(\lambda )=\frac{1-\lambda }{2\lambda }, \ \lambda \in (0,1)$, and $\eta =1e^{-3}$ with Momentum of 0.9 as the update step size rate. The hyperparameter $\lambda$ balances local and global information, affecting updates. Momentum-based approaches guide current upgrades using previous updates. Momentum component $q(\lambda )(v_{k,s-1}^{t-1} - w^{t-1})$ is also present. PIM $v_k^{t-1}$ is updated using information from the global model $w^{t-1}$. The momentum component’s weight is controlled by the hyper-parameter $\lambda$, operating between 0 and 1. PIM emphasizes global model recovery when $\lambda$ is around 0. It will align itself with the world data, to put it another way. Conversely, a greater focus on local training results from increasing $\lambda$. Table 2 shows the Hyperparameter settings for Federated Incremental Learning (FIL) experiments.

Analyze the impact that the samples have on the model’s result capacity for generalization to determine the significance of the samples, and provide more weight to those that may improve generalization. As the significance scores for samples, compute the gradient norm with regard to the PIM model parameters. Sample gradient norm during training may contribute to model updating. Identify significant scores for each local sample if on tasks $t-1$, client *k* has converged sample on the incremental *t*th position $T^{t-1}_{k,\text {local}}$. The sample’s gradient norm $(\tilde{x}^{(i)}_{k,t-1}, \tilde{y}^{(i)}_{k,t-1})$ during the PIM update in the *p*th iteration $v^{t-1}_{k,p}$ is represented here as $G_p(\tilde{x}^{(i)}_{k,t-1})$.

14 $$\begin{aligned} G^p\left( \tilde{x}_{k,t-1}^{(i)} \right) = \left\| \nabla \ell \left( f_{v_{k,p}^{t-1}}\left( \tilde{x}_{k,t-1}^{(i)} \right) , \tilde{y}_{k,t-1}^{(i)} \right) \right\| ^2 \end{aligned}$$

Equation (13) defines the sample gradient norm, which determines the upper limit on the incline of the difference between the loss function $(\tilde{x}_{k,t-1}^{(i)}, \tilde{y}_{k,t-1}^{(i)})$. The gradient may be minimized and training dynamics can be optimally maintained by caching samples according to sample gradient norms. Given that PIM incorporates both local and global models, a sample with a higher gradient norm driven by PIM is more likely to match the objective using both local and worldwide data. For PIM, where fluctuation near optima is uncommon, this impact could be more noticeable during *s* iterations, early training than it is later in the training process. In order to determine the sample significance, accumulate the gradient norm throughout PIM training, paying particular attention to the early training phase.

15 $$\begin{aligned} & I\left( \tilde{x}_{k,t-1}^{(i)} \right) = \sum _{p=1}^{S} \frac{1}{p} G^p\left( \tilde{x}_{k,t-1}^{(i)} \right) \end{aligned}$$

16 $$\begin{aligned} & w_{k,p}^t = w_{k,p-1}^{t} - \eta \sum _{i=1}^{M} \nabla \ell (f_{(w_{k,p-1}^{t}}, \tilde{x}_{k,t}^i), \tilde{y}_{k,t}^i) \end{aligned}$$

The local model $w_{k}^{t}$ is trained by each client using local samples $T_{k,\text {local}}^t$ in iteration $p \in [1, s]$ in the manner described below, following the caching of significant samples with higher significance scores. Algorithm 2 the convergence advantages and is given effect to the global model’s updating. Instead of needing additional extracted or produced data, which would increase compute or storage overhead, each client may use their local training data alone to develop a backbone model through FIL.

**Table 2:** Hyperparameter settings for federated incremental learning (FIL) experiments.

Parameter	Value / setting	Description
Momentum	0.9	Update step size rate to accelerate gradient descent.
Local Epochs (*E*)	5	Number of training passes over local data per round.
Batch size (*B*)	64	Number of samples per training batch.
Initial learning rate ($\eta$)	0.001	Learning rate used for all datasets at the start.
Training rounds	500	Total number of rounds for training across all datasets.
Regularization parameter ($\lambda$)	Initialized to 1; optimal value via grid search	Controls overfitting, tuned through grid search.

**Algorithm 2:**
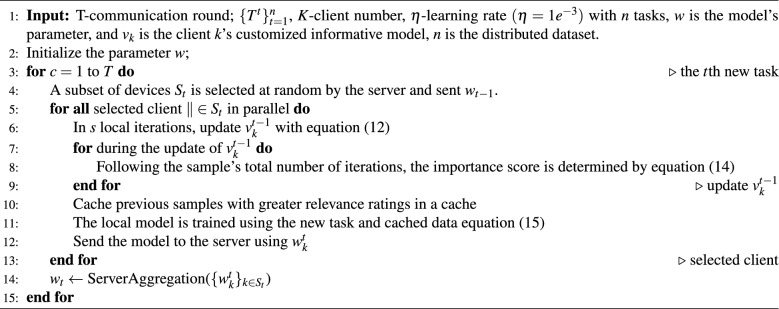
Federated incremental learning

#### Model initialization on blockchain

The blockchain creates and maintains the first machine learning model parameters, denoted by $M_0$, as part of providing the basis for collaborative learning by the model setup procedure. In particular, this step, $M_0 =\texttt {init()} \rightarrow \texttt {BChain}$ introduces the model and secures its parameters using the blockchain. The system prevents model development from being modified by documenting it on the blockchain from beginning to end, increasing security and confidence in collaborative learning. In a federated network, the basis for training is this initialization. As per the values of data integrity and decentralized trust, the framework offers a validated starting point by setting $M_0$ on the blockchain, which improves accountability and protects against unwanted changes. A transparent and secure method for restricting access to and modifications to data in FL settings is offered by blockchain technology. The FL process’s traceable and auditable record is produced via blockchain integration, which guarantees the results’ integrity and immutability. To make sure the data is not altered, the cryptographic hash value will be used to verify each block. The federated learning architecture is made more reliable by blockchain, which guarantees that both the training process and the prediction are unsolvable. The blockchain may be used to provide the transparency of an audit trail, allowing stakeholders to follow the model’s development and confirm its accuracy.

#### Local model training on devices

In the subsequent stage, each device restores the model with its individual own data via local model training. $M^\text {local}_{i+1} =\text {Train}(M_i, \text {localdata})$, where localdata $M_i$ is the global model’s parameters at iteration *I*, and the data is kept locally on each device. This architecture allows devices to train the model separately, protecting user privacy by doing away with the need to communicate raw data. Model flexibility is increased by local training, which allows each device to take use of distinct data features pertinent to its surroundings. FL primary objective is to train models in a decentralized manner while protecting data is reinforced by this locally executed stage, which gives the framework access to a variety of data sources without sacrificing privacy. With any quantity of input data, the cryptographic function known as Secure Hash Algorithm 256 (SHA-256) produces a 256-bit hash result. Each element of data on the blockchain has a unique hash generated by SHA-256. An input mapping to an output of fixed length *H*(*x*) is known as a hash function.^32^

17 $$\begin{aligned} H(x) = \text {SHA-256}(x) \end{aligned}$$

*x* is the block data in this case, which includes the hash of the previous block, its index, timestamp, and content. Its one-way function makes it collision resistant by guaranteeing that even some change in *x* results in an entirely new hash *H*(*x*). After encoding a block’s contents into JSON format, the compute hash function executes the SHA-256 algorithm and sorts the keys to preserve order.^32^

18 $$\begin{aligned} \text {Hash}_{\text {block}} = \text {SHA-256}(\text {JSON}(\text {block})) \end{aligned}$$

This protects data integrity across the blockchain by guaranteeing the uniqueness and immutability of each block’s hash^32^

#### Privacy preserving model

An adaptive privacy-preserving method based on local differential privacy (LDP) is suggested to safeguard the privacy of healthcare data while enhancing data usefulness to achieve an improved balance between security and functioning. Two or more malicious individuals work together in hidden obtaining other members personal data or targeting the global model in this attack scenario. Assume for the moment that an attacker engages in collusion assaults by controlling $f-1$ devices or *f* participants. The code, local models, and training datasets on $f-1$ devices have been revealed to the attackers. Specifically, under different situations, attackers may be aware of the aggregation rules^32^

#### Federated averaging with XAI integration

The framework modifies the FL procedure to maximize the influence of important characteristics by integrating XAI. The purpose of XAI is to prioritize changes and identify the significance of features, especially for sensor inputs that are prone to error. The contribution of each local model is weighed based on its determined significance to assist the XAI-driven insights. Following update cycle $i+1$, the model update from device K’s allocated weight may be shown as $W_{i+1}^k$. To continually improve the real-time information-based model, XAI creates the feedback loop is self-optimizing. This iterative process is shown by the following^32^

19 $$\begin{aligned} M_{i+1} = \frac{ \sum \limits _{k=1}^{N} W_{i+1}^k \left( M_{i+1}^k + \Delta M_\text {XAI}^k \right) }{ \sum \limits _{k=1}^{N} W_{i+1}^k } \end{aligned}$$

XAI-based modifications are included, enhancing the model’s robustness and security.

#### Blockchain validation and smart contract deployment

To ensure that it meets predefined accuracy criteria, the global model is evaluated. It is ensured by this process that only the best modifications to models are considered for deployment. An open record of every model version $M_{i+1}$ as $\text {Validate}(M_{i+1}) \rightarrow \text {B Chain}$ is provided by the blockchain, which renders the validated model indelible. By preventing unauthorized modifications and guaranteeing data integrity by only recording verified updates, the framework offers a powerful way to preserve trust in the FL process. The data is impenetrable because to consensus processes and hash functions, which prevent any individual from manipulating it. The ledger is represented by the chain of blocks $B = \{ b_1, b_2, \dots , b_n \}$, $i = 1,\dots ,n$, where $b_i$ is the *i*th block. Federated learning uses a global validation dataset to evaluate the performance (accuracy) of model after combining the updates from many clients^32^. The aggregated model so works effectively in a variety of data distributions. Instead of being approved in the distributed ledger, the validation condition makes sure that model modifications fulfill performance requirements. Hashing ensures the immutability and integrity of data. By ensuring that any alteration to a block makes the whole chain invalid, these restrictions protect the chain’s integrity.^32^

#### Consensus mechanism

A decentralized database dispersed over several network nodes is known as a distributed ledger. Cryptographically linked data is included in every block to guarantee tamper-proof storage. Federated averaging is used to aggregate the local updates from each node. To confirm modifications to the model from clients, establish confidence, and preserve the integrity of the distributed ledger, federated learning including blockchain requires a strong consensus mechanism. In the described setup, the consensus mechanism is the Proof of Contribution (PoC), compensating clients according on the way their model upgrades. AI systems that are secure, accessible and explicable are guaranteed by blockchain technology, application development responsibility and trust. Collaborative assaults, often referred to as collusion attacks, may be conducted by a number of malevolent actors in FBXAIO involving many parties. Two or more malicious individuals may work in hidden to obtain other members’ personal data or target the global model in this assault scenario.^32^.

#### EDBN detection and classification

To identify attacks and classify diseases from the secured dataset, EDBN is introduced in this study. Here, the number of layers and nodes which make each layer define the EDBN structure. As many layers and nodes are ideal for each dataset must thus be automatically determined to modify the structure. From this angle, potential DBN structures may be described as optimizing the space of solutions. Thus, with regard to a constraint condition and objective function, the issue for the generic optimization model may be mathematically represented as follows^32,33^.

20 $$\begin{aligned} \begin{aligned} \min _\text {s.t.} \;&f(x), {x \in X} \text { s.t.} \;&g_i(x) = 0, \; i = 1, \dots , n,&h_j(x) \le 0, \; j = 1,2, \dots , n \end{aligned} \end{aligned}$$

The equality constraints are represented by $g_i(x)$ and the inequality constraints by $h_j(x)$, respectively, whereas the target function is represented by $f(x)$. Thus, this is how the EDBN structure optimization model is constructed^32,33^

21 $$\begin{aligned} \min _\text {s.t.} R(C),{C \in \mathscr {C}} \; N_{\text {min}}(k) \le N_{\text {hid}}(k) \le N_{\text {max}}(k), \, \forall k \in \{1, \dots , n\}, \, D \le D_{\text {max}} \end{aligned}$$

All possible EDBN structures result in the solution space *C*, EDBN reconstruction error is denoted by *R*(*C*), and $k \in \{1 \ \text {to} \ 3\}$ is the index of the RBM in the EDBN, $N_{\text {hid}}(k)$ is the number of neurons in the hidden layer that *k*th RBM, and $N_{\text {min}}(k)$ and $N_{\text {max}}(k)$ are the hidden layer neurons with lowest and maximum levels respectively. *D*, $D_{\text {max}}$ is the depth, and maximum depth of DBN network^34^. Each of the two neighboring layers of neurons in an EDBN constitutes one RBM, which is composed of several layers of neurons. A visible layer and a hidden layer are created by the input and output of neurons. Neurons only connect to other neurons in the same layer; they do not connect to other neurons within the same layer. Based on the RBM that comes next, each layer of neurons could indicate up as a visible layer and a hidden layer, respectively. Consequently, a deep network with many RBMs stacked within it may be thought of as an EDBN. Its objective is to use network mapping; low-dimensional output vectors are used to represent high-dimensional input datasets. Data amount is measured by information entropy. It relates to the input dataset’s uncertainty in a physical sense. It is described as follows,^32,33^

22 $$\begin{aligned} H = \sum _{i=1}^{J} p(i) \log \left( \frac{1}{p(i)} \right) \end{aligned}$$

Where $H$ is the entropy of information, $J$ is sample count, and $p(i)$ is sample probability, where $\sum _{i=1}^{J} p(i) = 1$. Consider the number of visual layer nodes, $N_{vo}$, and the probabilities of the $i$th node’s state being zero or one, $p_i^0$ and $p_i^1$, respectively. The RBM visual layer’s information entropy $H_{vo}$ is computed using Eq. (23).^32^

23 $$\begin{aligned} H_{vo} = \sum _{i=1}^{No_{vo}} \left[ p_i(0) \log \frac{1}{p_i(0)} + p_i(1) \log \frac{1}{p_i(1)} \right] \end{aligned}$$

Additionally, let *Nohid* be the hidden layer’s number of nodes, $p_i(0)$ and $p_i(1)$ indicate the possibility that the $i^{\text {th}}$ node’s state is between 0 and 1, respectively, and $H_{hid}$ represent the entire data volume of the hidden layer, maximum value of *Hhid* is denoted as $H_{hid}^{max}$ arrives at $p_i(0) = p_i(1) = \frac{1}{2}$ with hidden layer neurons are limited to two states.^32^

24 $$\begin{aligned} H_{\text {hid}}^{\max } = \sum _{i=1}^{No_{\text {hid}}} \left[ -p_i^{\prime }(0) \log _2\left( p_i^{\prime }(0) \right) - p_i^{\prime }(1) \log _2\left( p_i^{\prime }(1) \right) \right] = \sum _{i=1}^{No_{\text {hid}}} \left[ \frac{1}{2} \log _2\left( \frac{1}{2} \right) + \frac{1}{2} \log _2\left( \frac{1}{2} \right) \right] = No_{\text {hid}} \end{aligned}$$

A maximum of information additional information than visible layer input data may be included in the hidden layer’s output vector. Information entropy determines the range of hidden layer node counts, which is $Hvo \le Nohid \le No0$. This is that the loss function is computed.^32,33^

25 $$\begin{aligned} L = \frac{1}{T} \sum _{t=1}^{T} \left[ \sum _{i=1}^{NO_{\text {vo}}} \sum _{j=1}^{No_{\text {hid}}} W_{ij} v_i(t) h_j(t) + \sum _{i=1}^{No_{\text {vo}}} a_i v_i(t) + \sum _{j=1}^{No_{\text {hid}}} b_j h_j(t) \right] \end{aligned}$$

*W* is the weight matrix, and *T* represents all of the sample training, and *L* is the loss function, $v_i(t)$ is the $i^{\text {th}}$ visible-layer neuron’s value, $h_j(t)$ is the $j^{\text {th}}$ hidden-layer neuron’s value, $a_i$ is the bias, and $b_j$ is the bias. Better network performance implies reduced loss.^32^

##  Results and discussion

The Python-based Tornado HTTP server communicates with Go’s native HTTP client to enable node-to-node communication. Datasets are used in experiments to compare with well-known FL schemes, including FedAvg, Privacy-Preserving Federated Blockchain Explainable Artificial Intelligence Optimization (PPFBXAIO), Federated Learning with Robust Aggregation in Edge Computing (FL-RAEC), Federated Learning with MultiParty Computation (FL-MPC), PEFL, and PPBEFL.The suggested model shows superior defense against a variety of assault concepts. Due to their complexity, wide range of features, and relevance to important healthcare predictions, these datasets are better suited for federated learning. FIL with batch size set to $B = 64$ by default, and local epochs set to $E = 5$. For every dataset, the starting learning rate $\eta$ is set at 0.001. Run models for 500 rounds on each dataset, respectively. Grid search is used to determine the ideal value of the regularization parameter $\lambda$, which is initially set to 1 by default (Refer Table 3).

**Table 3:** Experimental setup of simulation model.

Component	Specification/configuration
Hardware platform	NVIDIA RTX 4070 Ti GPU, 32 GB DRAM, Intel i5-13400 CPU with 10 cores at 2.50 GHz
Software environment	Python 3.10.11, PyTorch 2.0.1, Go HTTP client, Tornado HTTP server
Operating system	Microsoft Windows 10 professional (x64)
CPU, memory, graphics card	NVIDIA RTX 4070 Ti GPU, 32 GB DRAM, Intel i5-13400 CPU with 10 cores at 2.50 GHz
Federated learning framework	Flower (FLwr) for simulation and model distribution
Blockchain layer	Custom implementation using cryptographic SHA-256 and smart contract rules
Optimization algorithm	Chaotic Bobcat Optimization Algorithm (CBOA)
Model type	Entropy deep belief network (EDBN)
Privacy layer	RAPPOR-based local differential privacy (LDP)
Security mechanism	SHA-256 for hash computation and blockchain immutability
Consensus algorithm	Proof of contribution (PoC)
Train-test split	20% testing and 80% training
Metrics for evaluation	Accuracy, precision, recall, F-measure, loss, latency, and throughput
Attack models	Simulated DYN-OPT, STAT-OPT, label-flipping attack, and additional noise attack
Technology	Blockchain framework, federated learning

### Performance matrices

*Precision* Precision is mostly focused on the extent to which the model predicts positive results, which shows how well the model works when it makes a positive prediction,

26 $$\begin{aligned} \text {Precision} = \frac{TP}{TP + FP} \end{aligned}$$

*Recall* Recall is the total of the false negative and true positive forecasts divided by the number of true positive predictions. Recall gives information on how successfully a model detects every actual positive situation,

27 $$\begin{aligned} \text {Recall} = \frac{TP}{TP + FN} \end{aligned}$$

*F-Measure:* Harmonic mean of precision and recall known as F-Measure. It is computed by Eq. (28),

28 $$\begin{aligned} \text {F-Measure} = \frac{2 * \text {Precision} * \text {Recall}}{\text {Precision} * \text {Recall}} \end{aligned}$$

*Accuracy*: Accuracy is defined as the percentage of correctly predicted outcomes among all predictions.

29 $$\begin{aligned} \text {Accuracy} = \frac{\text {Number of Correct Predictions}}{\text {Total Predictions}} \times 100 \end{aligned}$$

*Loss*: The average local loss is calculated by aggregating the loss across all clients,

30 $$\begin{aligned} \text {Loss} = \frac{1}{N} \sum _{i=1}^{N} L_i \end{aligned}$$

where *N* stands for all of the clients, when the model’s output corresponds to the ground truth, this is referred to as “accurate prediction”.

*Latency*: Latency determines the amount of time needed to complete a system’s compute process or execute a request. The average of many rounds is often used to report it,

31 $$\begin{aligned} \text {latency}({ms}) = \frac{TT}{\text {Number of rounds}} \end{aligned}$$

Time is often measured in milliseconds (ms), where *TT* stands for the total time required for the procedure.

*Throughput*: Throughput measures how rapidly changes or transactions are processed by the system,

32 $$\begin{aligned} \text {Throughput} = \frac{\text {Transactions processed in total}}{TT} \end{aligned}$$

The total number of transactions processed is the amount of data packets that the system manages during the test period.

###  Performance comparison with other models

**Fig. 6:**
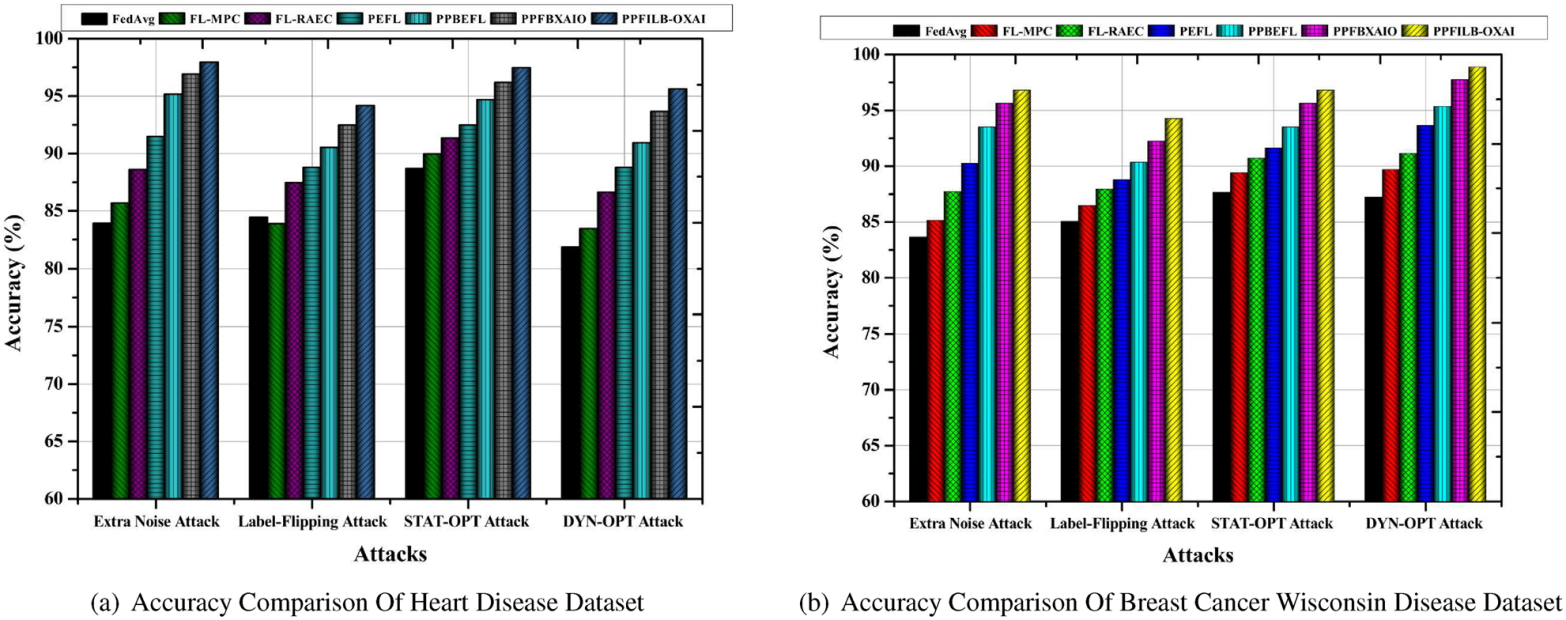
Comparison of dataset accuracy using FL techniques to prevent attacks.

The accuracy comparison of common FL schemes against different attack models like Extra Noise Attack^35^, Label-Flipping Attack^36^, Static Optimization (STAT-OPT) Attack^37^, and Dynamic Optimization (DYN-OPT) Attack^38^ is shown in Fig. 6 a, b.

The proposed approach achieves the greatest accuracy results of 97.93% and 96.77% against additional noise attack in heart disease and breast cancer dataset as shown in Fig. 6 a, b. Additionally, compared to other techniques, it may also provide more accurate findings for other attack. The lowest accuracy findings for heart disease are 83.93%, 85.69%, 88.61%, 91.45%, 95.13%, and 96.87% for existing methods when tested against additional noise attack. The lowest accuracy scores for breast cancer wisconsin are 83.61%, 85.15%,87.67%, 90.24%, 93.48%, and 95.63% for existing methods when tested against additional noise attack.

**Fig. 7:**
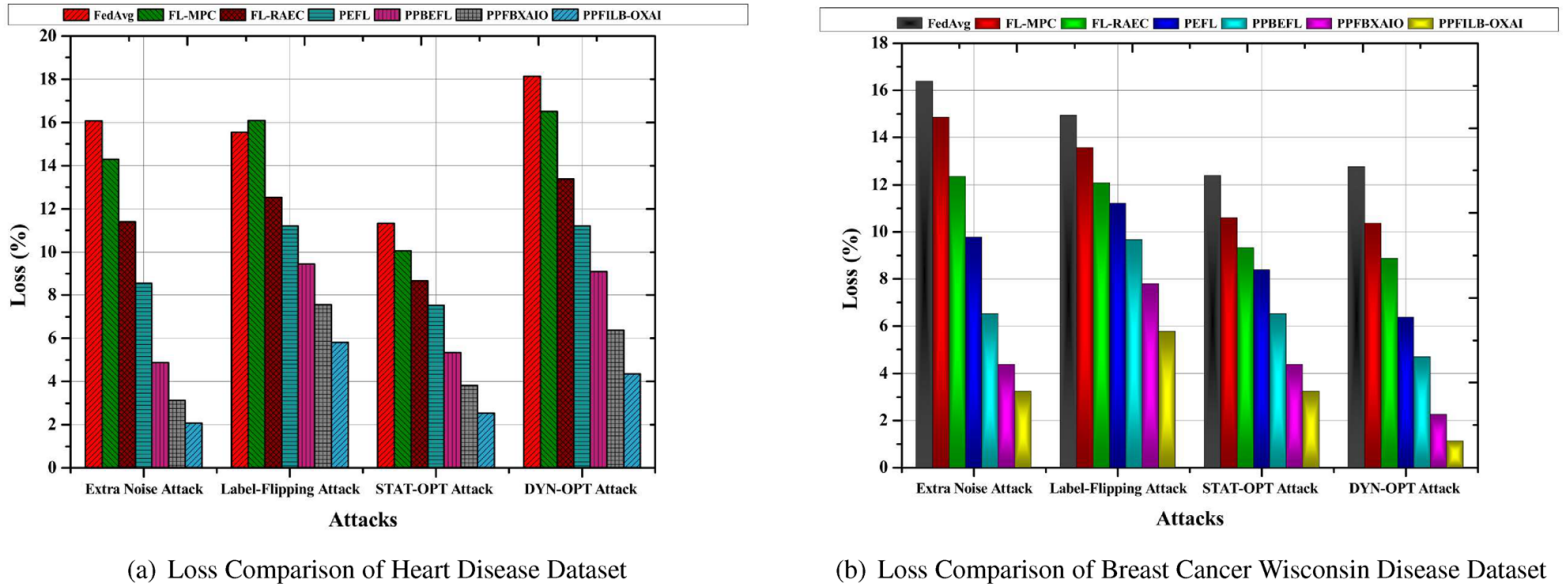
Comparison of dataset losses using FL techniques to prevent attacks.

Figure 7 a, b shows the proposed model has lowest loss of 2.07% and 3.23% for the heart disease dataset and breast cancer wisconsin dataset against additional noise attack. The enhanced loss results for heart disease provided by existing methods against additional noise attack are 16.07%, 14.35%, 11.39%, 8.55%, 4.87%, and 3.13%, respectively. Increased loss results for breast cancer dataset are 16.39%, 14.85%, 12.33%, 9.76%, 6.52%, and 4.37% when existing methods are used to excessive noise attack.

**Fig. 8:**
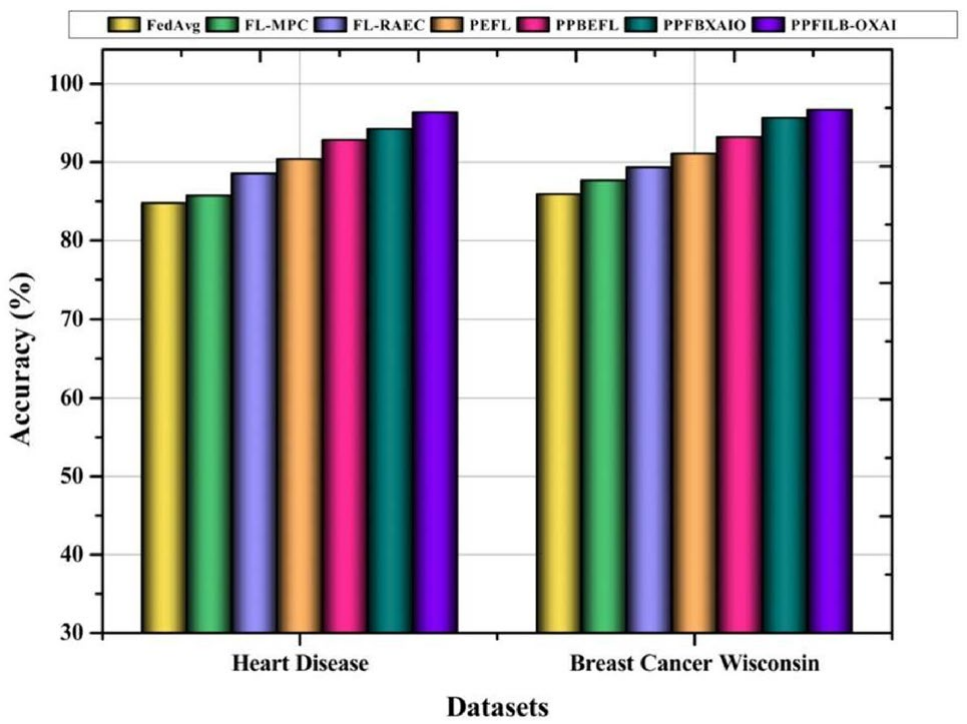
Comparing dataset accuracy with FL methods.

Figure 8 shows that the recommended method produces the highest accuracy results for heart disease,and breast cancer datasets with 96.30% and 96.66%, respectively. The lowest accuracy results for breast cancer wisconsin are provided by existing methods, which are 85.88%, 87.66%, 89.35%, 91.07%, 93.16%, and 95.59%, respectively.

**Fig. 9:**
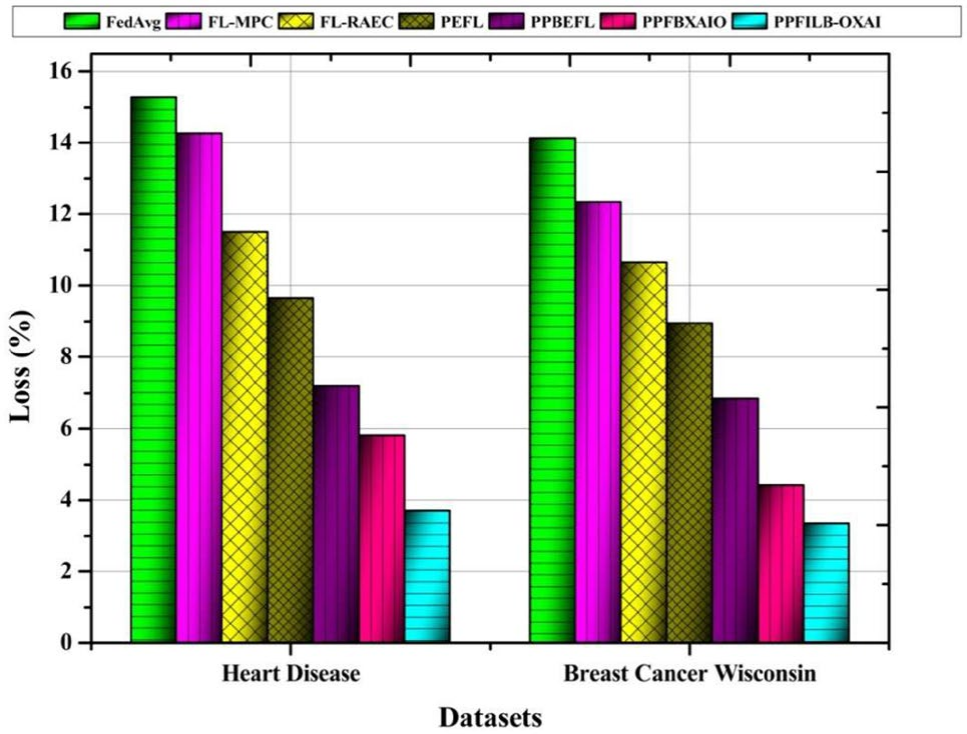
Comparing dataset losses with FL methods.

Figure 9 illustrates how the suggested approach provides the lowest loss results for heart disease, and breast cancer datasets at 3.34% and 3.70%, respectively. In a similar vein, it may also provide better outcomes than other techniques for different assaults. The lowest loss results for heart disease are provided by existing methods, which are 15.27%, 14.25%, 11.49%, 9.63%, 7.19%, and 5.81%, respectively. For breast cancer dataset, the highest loss results are 14.12%, 12.34%, 10.65%, 8.93%, 6.84%, and 4.41% for existing methods.Table 4 shows the comparison of FL techniques’ performance compared to Sybil, and collision attacks against accuracy, and loss. Proposed model has highest accuracy of 96.46%, and 93.67% against Sybil, and collision attacks in Heart disease dataset. Proposed model has lowest loss of 3.54%, and 6.33% against Sybil, and collision attacks in Heart disease dataset.

**Table 4:** Results comparison of federated learning (FL) methods under Sybil and collision attacks.

Dataset	Methods	Accuracy (%)		Loss (%)	
		Sybil	Collision	Sybil	Collision
Heart disease	FedAvg	83.11	82.54	16.89	17.46
	FL-MPC	85.24	83.46	14.76	16.54
	FL-RAEC	87.88	85.69	12.12	14.31
	PEFL	90.25	87.38	9.75	12.62
	PPBEFL	93.39	89.57	6.61	10.43
	PPFBXAIO	94.93	91.28	5.07	8.72
	PPFILB-OXAI	96.46	93.67	3.54	6.33
Breast cancer wisconsin	FedAvg	82.62	81.41	17.38	18.59
	FL-MPC	84.47	83.66	15.53	16.34
	FL-RAEC	86.78	85.83	13.22	14.17
	PEFL	89.09	87.56	10.91	12.44
	PPBEFL	92.94	90.18	7.06	9.82
	PPFBXAIO	94.17	91.61	5.83	8.39
	PPFILB-OXAI	96.36	93.75	3.64	6.25

### Comparison of disease prediction methods’ performance

FedHFP+RNN, FedHFP+LSTM, DeFedHDP+EDBN, FedAvgBC+TabNet, PPFBXAIO+EDBN, and proposed model compared using metrics including recall, precision, f-measure, and accuracy. These techniques are used in Wisconsin to treat breast cancer and heart problems, including a number of health-related features of the patients. An 80-20 train-test split was used to confirm the classifiers efficiency. Table 5 shows the accuracy results comparison of FL methods against feature selection methods. It shows that the proposed CBOA selection methods has highest accuracy of the Wisconsin datasets for breast cancer and heart disease were 95.71% and 96.84%, respectively.

**Table 5:** Accuracy comparison of disease prediction methods using feature selection (FS) methods.

Dataset	Methods	SelectKBest	Lasso	Recursive feature elimination(RFE)	CBOA
Heart disease	FedHFP+RNN	80.31	82.45	83.33	85.15
	FedHFP+LSTM	81.78	83.64	85.39	86.79
	DeFedHDP+EDBN	84.25	85.80	87.03	88.78
	FedAvgBC+TabNet	85.87	87.56	89.21	91.41
	PPFBXAIO+EDBN	87.79	89.39	90.74	93.07
	PPFILBOXAI+EDBN	89.87	91.66	92.79	95.71
Breast cancer wisconsin	FedHFP+RNN	81.56	83.54	84.31	85.58
	FedHFP+LSTM	82.95	84.81	85.96	87.17
	DeFedHDP+EDBN	84.38	86.72	87.90	89.28
	FedAvgBC+TabNet	86.62	88.25	89.83	92.44
	PPFBXAIO+EDBN	88.77	90.50	91.58	95.07
	PPFILBOXAI+EDBN	91.05	92.36	93.75	96.84

**Fig. 10:**
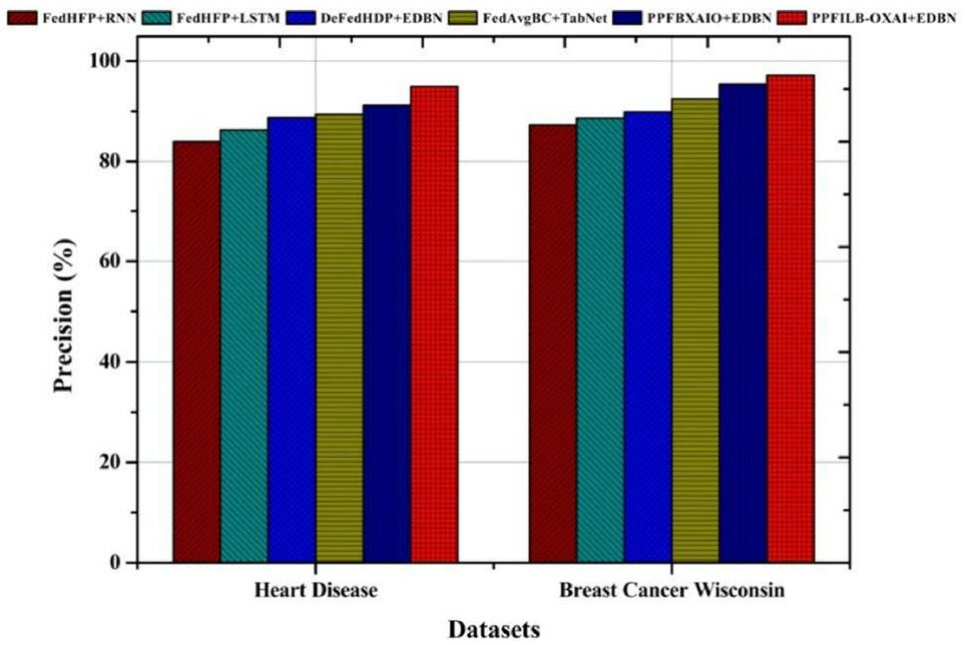
Comparison of prediction models in terms of precision.

Precision comparisons of prediction methods are shown in Fig. 10. The suggested classifier precision values for heart disease, and breast cancer datasets are 97.13% and 94.87% respectively. The precision results for heart disease provided by existing methods were 83.85%, 86.25%, 88.68%, 89.31%, and 91.19%, respectively. The precision results for breast cancer dataset are 87.21%, 88.60%, 89.77%, 92.42%, and 95.44% for methods. Because the features in the dataset are optimally selected, PPFBXAIO+EDBN have the greatest accuracy.

**Fig. 11:**
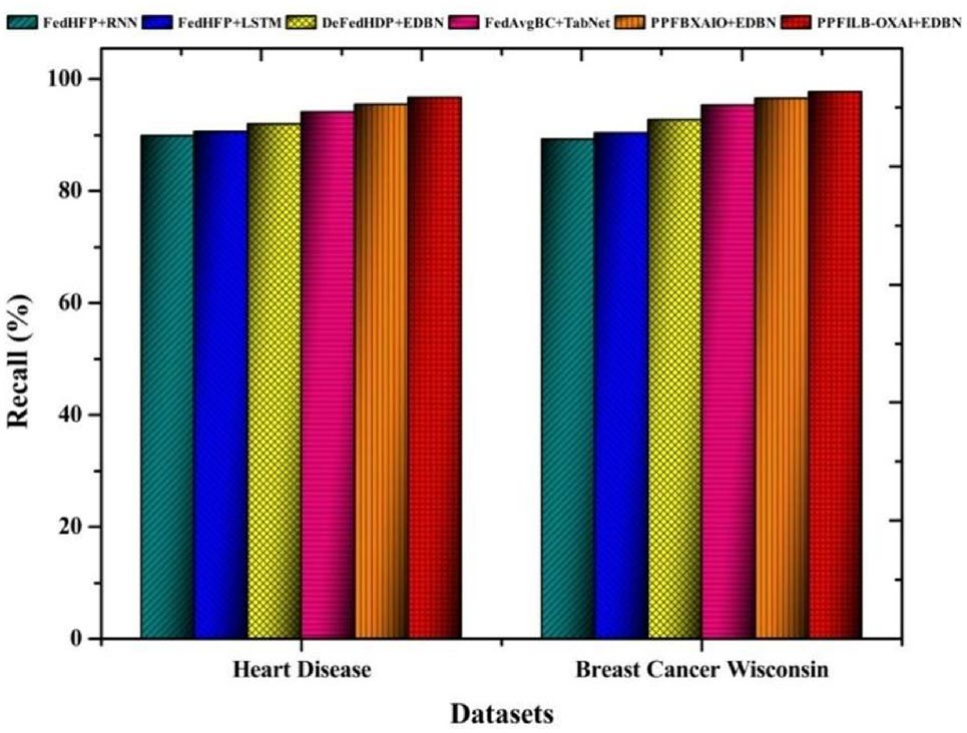
Comparison of prediction models in terms of recall.

Classifiers all show the recall contrast in Fig. 11. For heart disease, and breast cancer dataset, the proposed classifier has the greatest recall rates, 96.73% and 97.70%, respectively. Recall values for heart disease are 89.86%, 90.60%, 92.00%, 94.04%, and 95.39% for classifiers. Recall results for breast cancer wisconsin are according to classifiers as 89.24%, 90.41%, 92.66%, 95.36%, and 96.54%.

**Fig. 12:**
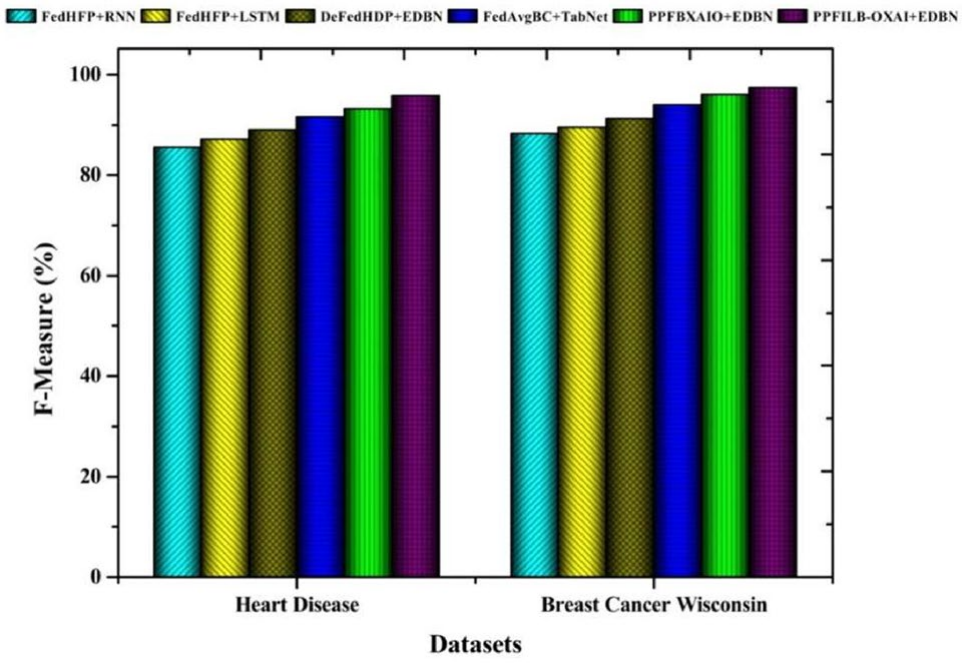
Comparison of prediction models in terms of F-measure.

Classifiers with respect to f-measure comparisons are shown in Fig. 12. Heart disease, and breast cancer wisconsin datasets had the highest f-measure results for the proposed classifier, 95.79% and 97.41%, respectively. The f-measure results for heart disease are 85.53%, 87.09%, 89.03%, 91.61%, and 93.24% for classifiers. The f-measure values for breast cancer dataset according to classifiers are 88.21%, 89.49%, 91.20%, 93.87%, and 95.98%.

**Fig. 13:**
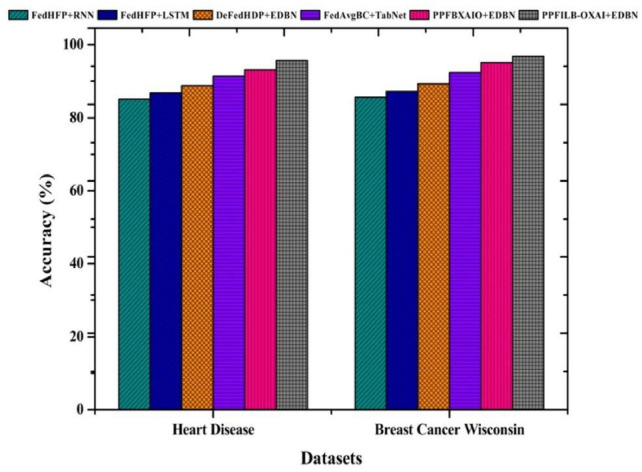
Comparison of various prediction models’ accuracy.

Classifiers accuracy comparisons are shown in Fig. 13. The highest accuracy values for heart disease, and breast cancer wisconsin are 95.71% and 96.84%, respectively, using the proposed classifier. For heart disease, the accuracy findings from classifiers are 85.15%, 86.79%, 88.78%, 91.41%, and 93.07%, respectively. For breast cancer wisconsin, the accuracy findings for classifiers are 85.58%, 87.17%, 89.28%, 92.44%, and 95.07%, respectively.

**Fig. 14:**
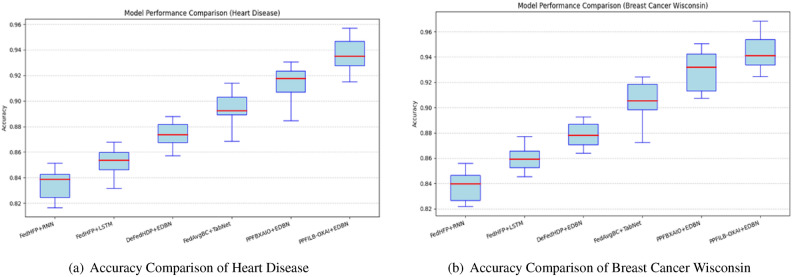
Accuracy comparison of Boxplots for FL methods.

Classifiers accuracy comparison with boxplots against FL methods is shown in Fig. 14 . The highest accuracy values for heart disease, and breast cancer wisconsin are 95.71% and 96.84%, respectively, using the proposed classifier. For heart disease, the accuracy findings from classifiers are 85.15%, 86.79%, 88.78%, 91.41%, and 93.07%, respectively. For breast cancer wisconsin, the accuracy findings for classifiers are 85.58%, 87.17%, 89.28%, 92.44%, and 95.07%, respectively.

### Metrics comparison with other models

Typical FL schemes are compared in this section in terms of latency, throughput, and computational overhead vs. the number of rounds ranging from 25 to 100.

**Fig. 15:**
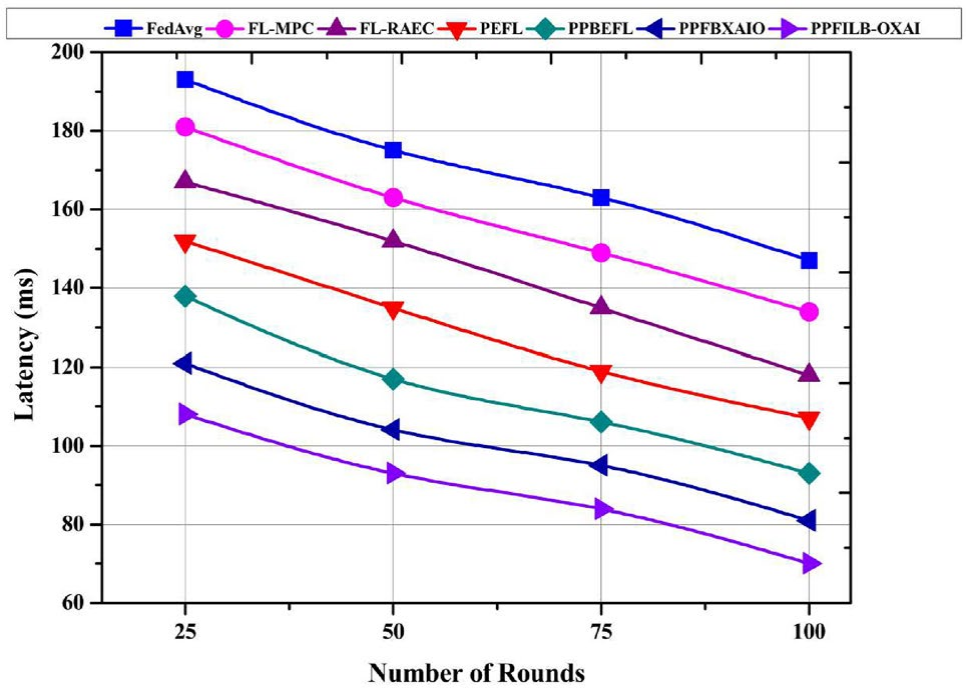
Comparison of FL methods latency.

The latency of FL techniques is compared with the number of rounds from 25 to 100 with a 25-round gap in Fig. 15. PPFBXAIO system has 108 ms, 93 ms, 84 ms, and 70 ms for 25, 50, 75, and 100 rounds are the lowest latency results. The increased latency results for 100 rounds were 147 ms, 134 ms, 118 ms, 107 ms, 93 ms and 81 ms for methods. Compared to existing approaches, the PPFILB-OXAI system has a lower latency at 77 ms, 64 ms, 48 ms, 37 ms, 23 ms and 11 ms.

**Fig. 16:**
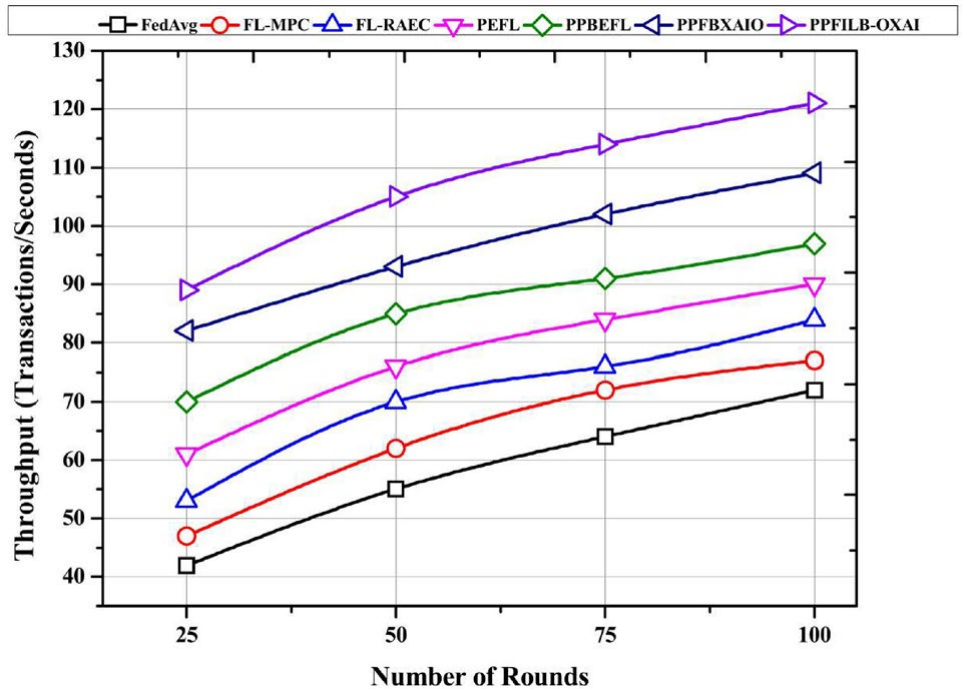
Comparison of FL methods by throughput.

A comparison of privacy-preserving approaches’ throughput by number of rounds is shown in Fig. 16. PPFILB-OXAIO system has 89, 105, 114, and 121 transactions/second for 25, 50, 75, and 100 rounds are the greatest throughput. FedAvg simple design contributed to its lowest results of 72 transactions per second, but it didn’t enhance blockchain dependability. Throughputs of 77 and 84 transactions/second are achieved by FL-MPC and FL-RAEC, respectively. Results from PEFL, PPBEFL, and PPFBXAIO are 90, 97, and 109 transactions/second respectively.

**Fig. 17:**
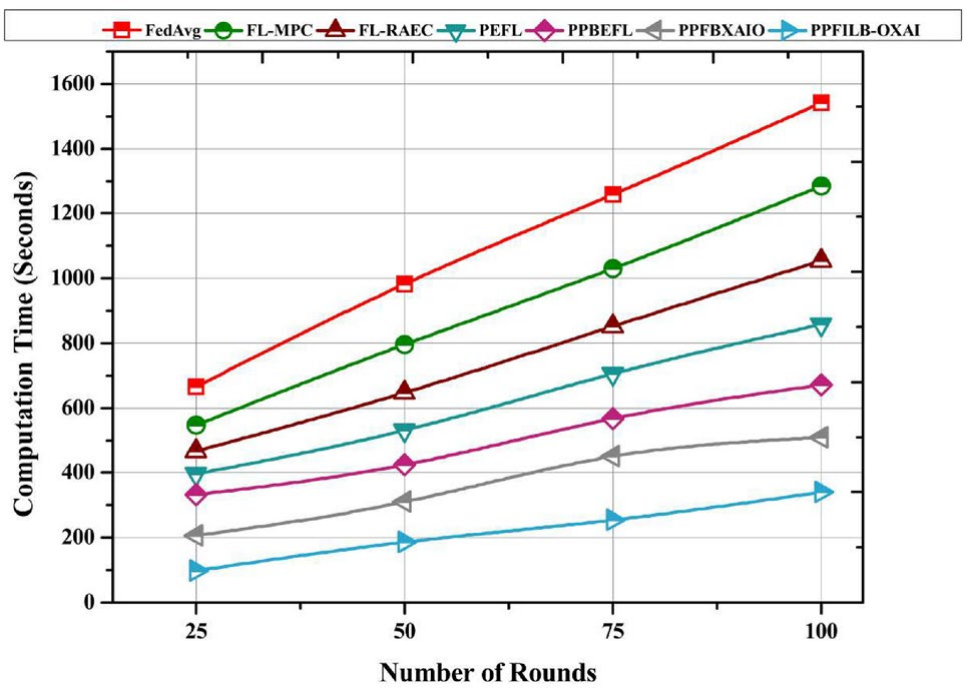
Computational time comparison for FL methods.

Figure 17 shows a comparison of the FedAvg, FL-MPC, and FL-RAEC execution times. As the number of rounds developed, all models showed an increase in computational cost. However, PPFILB-OXAI model had a significantly lower computational time of 341 seconds, existing methods has increased computational time of 1541 seconds, 1285 seconds, 1054 seconds, 858 seconds, 671 seconds, and 509 seconds. The suggested method reduces verification with conventional encryption and decryption procedures. By contrast, FL methods that rely on decryption for verification run the risk of squandering time in the event that verification is failed.

**Fig. 18:**
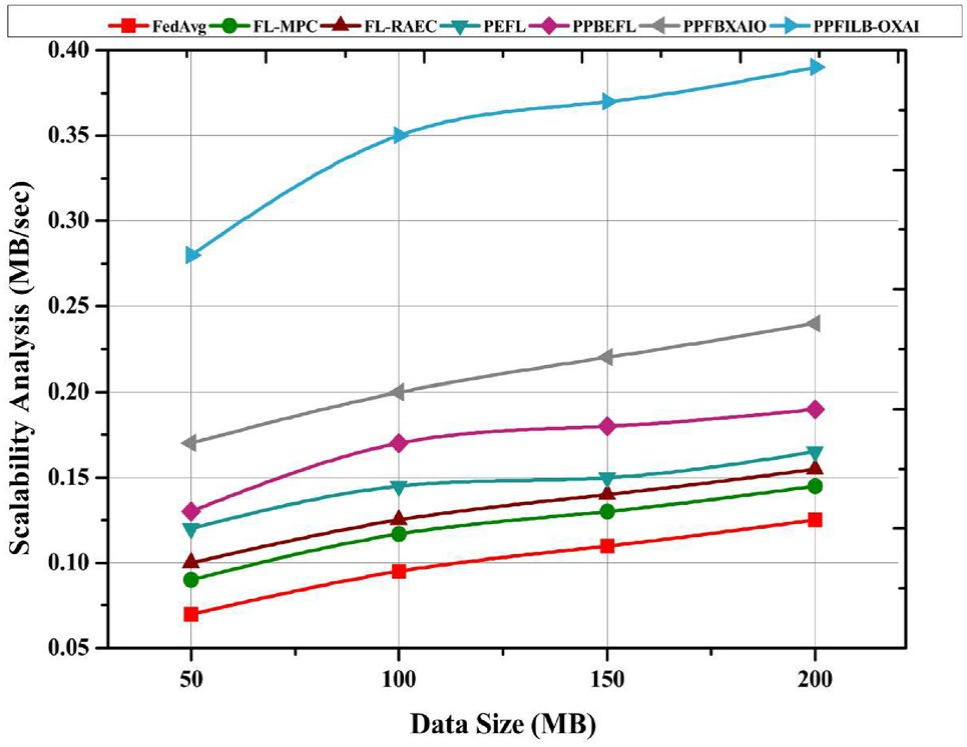
Scalability comparison for FL methods.

Figure 18 shows a scalability comparison of the FL methods with respect to data size. PPFILB-OXAI model had a significantly increased scalability analysis of 0.392 MB/seconds, other methods such as FedAvg, FL-MPC, FL-RAEC, PEFL, PPBEFL, PPFBXAIO have a decreased scalability analysis of 0.124MB/seconds, 0.142MB/seconds, 0.156MB/seconds, 0.168MB/seconds, 0.182MB/seconds and 0.231 MB/seconds.

**Fig. 19:**
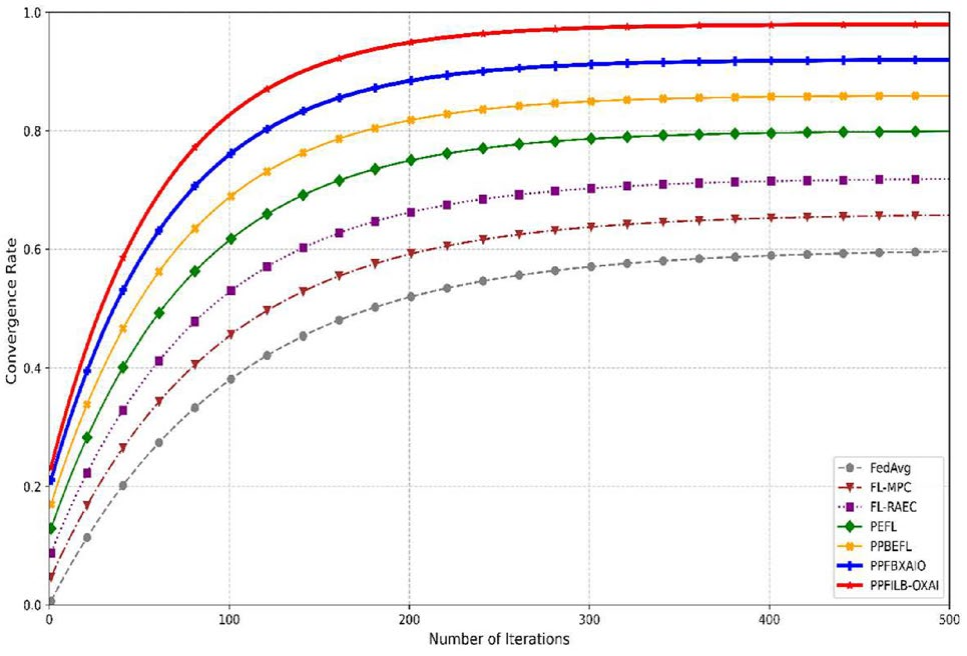
Convergence rate comparison for FL methods.

Figure 19 shows a convergence rate comparison of the FedAvg, FL-MPC, FL-RAEC, PEFL, PPBEFL, PPFBXAIO, and PPFILB-OXAI with respect to number of iterations 100–500. For increased iterations proposed system has increased convergence rate upto 0.98 saturated value for 300, 400, 500 iterations which is increased than the other methods.

**Fig. 20:**
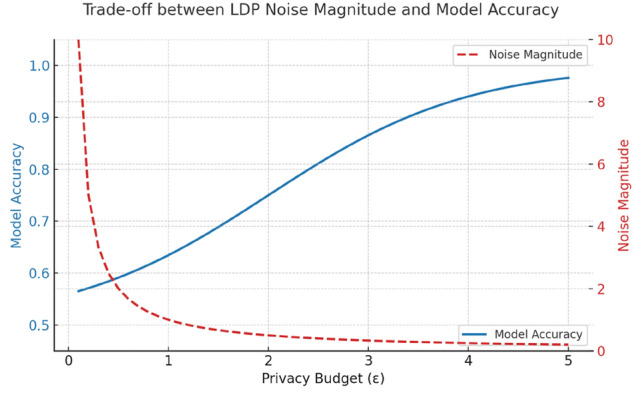
Trade-off between Ldp noise magnitude and accuracy of model.

Figure 20 shows a trade-off evaluation of the suggested model in context of respect to privacy (1-5). For increased privacy, the accuracy of the suggested method has grown to 0.95. Lowest privacy level, proposed system has lowest accuracy of 0.56, privacy increases with increased accuracy. It shows that the proposed system is not easily violated by attacks in the model. Proposed model achieved a highest results of p<0.001 which is more significant in privacy preservation.

Paired t-tests comparison of PPFILB-OXAI method with respect to different traditional methods shows highly significant p-values (all p< 0.001) signifying that the performance differences are statistically significant.

**Table 6:** Paired t-tests comparison of FL methods.

Method	t-statistic	*p*-value	Significance
FedAvg	43.86	$8.32 \times 10^{-12}$	Highly significant
FL-MPC	38.48	$2.69 \times 10^{-11}$	Highly significant
FL-RAEC	29.12	$3.23 \times 10^{-10}$	Highly significant
PEFL	67.08	$1.84 \times 10^{-13}$	Highly significant
PPBEFL	27.52	$5.36 \times 10^{-10}$	Highly significant
PPFBXAIO	4.94	$8.04 \times 10^{-14}$	Statistically significant
PPFILB-OXAI	2.19	$3.64 \times 10^{-16}$	Highly statistically significant

Here the FL methods, including FedAvg, FL-MPC, FL-RAEC, PEFL, PPBEFL, and PPFBXAIO were compared by the PPFILB-OXAI in healthcare. Table 6 demonstrates that the suggested strategy performs significantly more effectively in terms of accuracy than other FL methods, according to a paired t-test. Consistent progress is shown throughout all test runs in the linked accuracy information. When compared to other FL approaches, PPFILB-OXAI has a more focused (less variation) and greater accuracy distribution according to boxplot analysis. Line plots linking matched accuracy observations clearly show that PPFILB-OXAI consistently beats the accompanying FL technique run, demonstrating clear benefits in every single case.

##  Conclusion and future work

In this paper, PPFILB-OXAI is introduced which leveraging the benefits Blockchain, Federated Incremental Learning (FIL), and explainable artificial intelligence (XAI) with optimization. Initially, dataset has been collected from Kaggle. The original data performs linear transformation and min-max normalization. CBOA technique is used to update the population in feature selection and it is inspired by bobcats typical hunting strategies. First, the bobcat follows its prey and then approaches it using this method. CBOA is performed on continuously until a certain number of repetitions are discovered than the worst person in the population. FIL cases when customers lack room to save all data. Consider about two IL scenarios: (2) domain incremental task, all tasks share domain space each task has the same classes. Then, each client is in control of both the local and global models initially trains a more customized informative model on previous local samples when new tasks arise. For the FIL in blockchain, PPFILB-OXAI is introduced, which is required for the exchange of sensitive data encrypted using Secure Hash Algorithm 256 (SHA-256). Performance criteria including accuracy, precision, recall, and F-measure show that the proposed system outperforms alternative approaches. The use of hybrid approaches in the healthcare area is not included in the existing set of work. Furthermore, accuracy is compromised when privacy-preserving procedures are used. When using AI in the healthcare industry, accuracy is an essential problem. The computation cost required to generate random numbers might cause the execution time to delay. Compared to plaintext computing, secret sharing requires more communication and connection between all parties, which increases the communication costs.

## Data Availability

The data that support the findings of this study are available from the corresponding author, upon reasonable request.
